# Luminal Rank loss decreases cell fitness leading to basal cell bipotency in parous mammary glands

**DOI:** 10.1038/s41467-023-41741-5

**Published:** 2023-10-09

**Authors:** Ana Sofia Rocha, Alejandro Collado-Solé, Osvaldo Graña-Castro, Jaime Redondo-Pedraza, Gonzalo Soria-Alcaide, Alex Cordero, Patricia G. Santamaría, Eva González-Suárez

**Affiliations:** 1 Oncobell, Bellvitge Biomedical Research Institute, IDIBELL, Barcelona, Spain; 2 Molecular Oncology, Spanish National Cancer Research Center (CNIO), Madrid, Spain; 3 Bioinformatics Unit, Spanish National Cancer Research Center (CNIO), Madrid, Spain; 4 Department of Basic Medical Sciences, Institute of Applied Molecular Medicine (IMMA-Nemesio Díez), School of Medicine, San Pablo-CEU University, CEU Universities, Boadilla del Monte, Madrid, Spain

**Keywords:** Mammary stem cells, Cell lineage, Cell signalling

## Abstract

Rank signaling pathway regulates mammary gland homeostasis and epithelial cell differentiation. Although Rank receptor is expressed by basal cells and luminal progenitors, its role in each individual cell lineage remains unclear. By combining temporal/lineage specific Rank genetic deletion with lineage tracing techniques, we found that loss of luminal Rank reduces the luminal progenitor pool and leads to aberrant alveolar-like differentiation with high protein translation capacity in virgin mammary glands. These Rank-deleted luminal cells are unable to expand during the first pregnancy, leading to lactation failure and impairment of protein synthesis potential in the parous stage. The unfit parous Rank-deleted luminal cells in the alveoli are progressively replaced by Rank-proficient cells early during the second pregnancy, thereby restoring lactation. Transcriptomic analysis and functional assays point to the awakening of basal bipotency after pregnancy by the induction of Rank/NF-κB signaling in basal parous cell to restore lactation and tissue homeostasis.

## Abstract (web summary)

Rocha and co-authors show that loss of luminal Rank signaling causes abnormal alveolar differentiation and lactation failure. Subsequent pregnancies activate bipotency in basal cells, replacing unfit luminal cells, and restoring lactation.

## Introduction

The mammary gland (MG) is a highly specialized organ that primarily develops after birth, undergoing expansion during puberty, followed by cycles of growth and regression with each oestrous and pregnancy/lactation/involution cycle. The MG epithelium is organized as a bilayer composed of luminal cells, lining the ductal or alveolar lumen, and basal cells surrounding the luminal layer. The luminal compartment is further subdivided into hormone-sensing cells, expressing hormone receptors (HRs) and luminal progenitors lacking HRs.

Tissue homeostasis is maintained by at least two subsets of lineage-restricted progenitors responsible for the maintenance and expansion of each of the cellular compartments independently (reviewed in^1^). The basal lineage, long recognized to be enriched in stem cells, although also heterogeneous, has not been extensively characterised due to the lack of markers discriminating putative stem cells from the more differentiated counterparts.

Rankl (receptor activator of nuclear factor-B ligand), a cytokine member of the TNF superfamily, and its receptor Rank are essential for MG development^2,3^. In the context of the mouse epithelial hierarchy, Rankl is expressed in progesterone receptor-positive (PR+) luminal cells, mediating progesterone dependent mitogenic signaling in Rank-positive neighbouring cells, namely luminal progenitors and basal cells^4–6^. Full body and epithelial specific Rank deletion display defective alveologenesis and lactation failure due to impaired mammary epithelial cell (MEC) proliferation and differentiation^2,6^, while pharmacological inhibition (Rank-Fc treatment) at mid-pregnancy results in premature lactogenesis through the interference with prolactin/Stat5 signaling^7^. Moreover, MMTV-driven Rank expression in the MG results in increased proliferation and stemness, accompanied by early alveologenesis but failure to lactate^8–10^.

Rank pathway signaling is therefore required for epithelial expansion and alveologenesis, while its downregulation is fundamental to achieve terminal differentiation and milk production. Although the consequences of full body or constitutive epithelial Rank deletion have been established^2,6^, the contribution of Rank signaling to each mammary cell lineage has not been addressed. Here, we show that Rank basal cell specific deletion has a minor impact in MG homeostasis. In contrast, luminal Rank signaling has a crucial role in the maintenance of luminal progenitors by preventing precocious alveolar differentiation and renewal during lactation/regression cycles. Finally, we demonstrate that under physiological stress driven by dysfunctional luminal cells, basal cell bipotency is activated through Rank signaling, thus contributing to MG homeostasis.

## Results

### Luminal Rank deletion leads to an increase in hormone-sensing population without affecting fat pad invasion

**Fig. 1:**
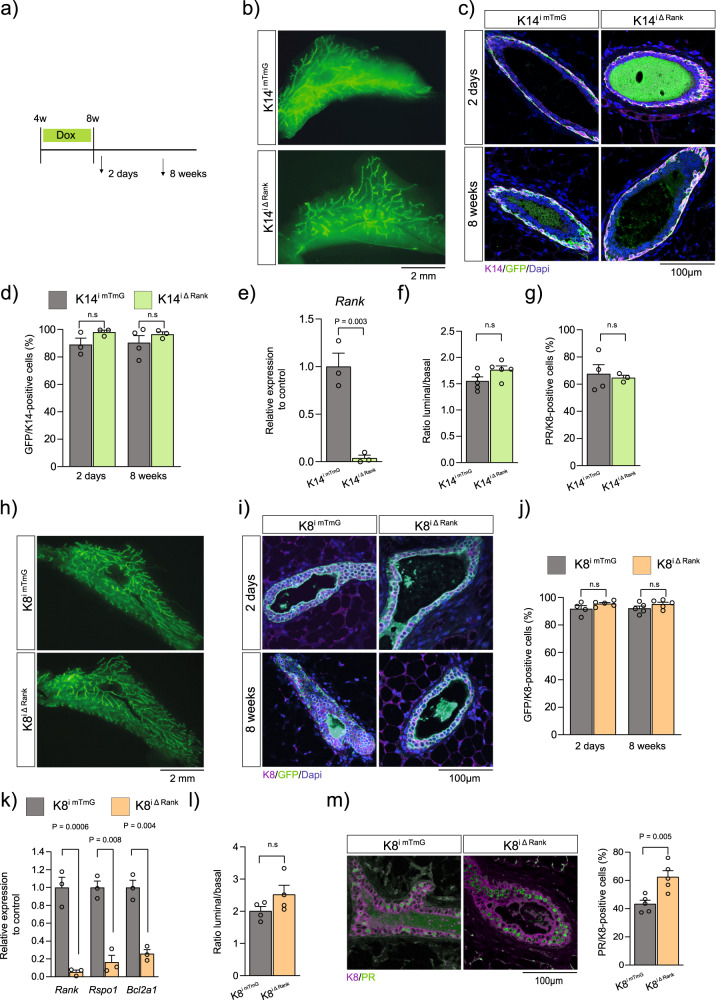
Luminal Rank deletion reduces luminal progenitors without altering fat pad invasion. **a** Experimental protocol used to achieve complete Rank deletion in virgin MGs from K14 and K8 transgenic mouse models (for details see methods). **b** Whole mount IF (GFP) from K14^imTmG^ and K14^iΔRank^ MGs 2 days following doxycycline (dox) removal. **c** Images from IF detecting K14+ (magenta) and GFP+ (green) cells, Dapi (blue) stains nuclei. **d** Quantification of GFP+ cells in the K14 population in K14^imTmG^ and K14^iΔRank^ mice 2 days (*n* = 3; *p* = 0.30) and 8 weeks (*n* = 4 K14^imTmG^; *n* = 3 K14^iΔRank^; *p* = 0.51) post-dox removal. **e** qPCR of *Rank* mRNA in FACS-sorted basal cells from K14^iΔRank^ MGs relative to controls (*n* = 3; *p* = 0.003). **f** Ratio of luminal (K8+) to basal (K14+) cells based on IF analysis 8 weeks following dox removal from K14^imTmG^ and K14^iΔRank^ MGs (*n* = 5; *p* = 0.08). **g** Quantification of PR+ within luminal cells (K8+) 2 days following dox removal from K14^imTmG^ (*n* = 4) and K14^iΔRank^ (*n* = 3) mice (*p* = 0.73). **h** Whole mount IF (GFP) from K8^imTmG^ and K8^iRank^ MGs 2 days after dox removal. **i** IF images of K8+ (magenta) and GFP+ (green) cells in indicated MGs, Dapi (blue) stains nuclei. **j** Quantification of GFP+ cells in the K8+ population 2 days (*n* = 4 K8^imTmG^; *n* = 5 K8^iΔRank^; *p* = 0.25) and 8 weeks (*n* = 5; *p* = 0.34) following dox removal. **k** qPCR of *Rank* (*p* = 0.0006), *Rspo1* (*p* = 0.008)*, Bcl2a1* (*p* = 0.004) in FACS-sorted luminal GFP+ cells from K8^imTmG^ and K8^iΔRank^ mice (*n* = 3). **l** Ratio of luminal (K8+) to basal (K14+) cells in K8^imTmG^ and K8^iΔRank^ MGs 2 days following dox removal (*n* = 4; *p* = 0.16). **m** IF and quantification of PR+ (green) within luminal K8+ (magenta) cells 8 weeks following dox removal (*n* = 5; *p* = 0.005). Data are represented as mean +/− SEM. Scale bars and significant *P*-values are indicated in the graphs. *P*-values were calculated by Two-Way ANOVA with Tukey’s multiple comparisons (**d**, **j**), Unpaired *T*-test two-tailed (**e**, **f**, **g**, **l**, **m**) and One Sample *T*-Test (**e**, **k**). Staining was quantified in 5 independent images from two tissue sections collected 100 µm apart (**d**, **j**, **m**). Source data are provided as a Source Data file. ns not significant.

Rank has been shown to be expressed in basal cells of the MG epithelium and, at lower levels, in luminal progenitors (HR−)^11^. We thus aimed to determine the role of Rank in each of these cell lineages independently. We induced Rank deletion during puberty (Fig. 1a) to allow fate mapping of the recombined cells (GFP+) at the different stages in K14^iΔRank^ (targeting basal cells), K8^iΔRank^ (targeting luminal cells) and control mouse lines lacking the Rank floxed allele but including the mTmG reporter (K14^imTmG^ and K8^imTmG^) (Supplementary Fig. 1a, b).

Whole mount analysis of K14^iΔRank^ MGs did not show major defects in fat pad invasion upon basal Rank loss (Fig. 1b), despite the high levels of recombination that remained stable throughout the tracing in K14^iΔRank^ MGs (Fig. 1c, d and Supplementary Fig. 1c). Furthermore, qPCR on flow cytometry sorted-GFP basal cells confirmed efficient *Rank* deletion in this population (Fig. 1e). Histological analysis demonstrated a normal distribution of luminal (K8+) and basal (K14+) populations (Figs. 1c, 1f and Supplementary Fig. 1d), as well as proportion of PR+ cells within the luminal epithelium (Fig. 1g).

An analogous approach was performed in K8^iΔRank^ MGs; again, we were unable to detect any major alterations in fat pad invasion (Fig. 1h). High efficiency and specificity of recombination in luminal cells were confirmed by immunofluorescence (IF) and flow cytometry (Fig. 1i, j and Supplementary Fig. 1e, f). A clear reduction in *Rank* gene expression and its downstream targets in K8^iΔRank^ sorted-luminal cells confirmed efficient luminal Rank deletion (Fig. 1k). Histological analysis failed to demonstrate an overt phenotype in K8^iΔRank^ mice and no difference in the ratio luminal/basal cell was detected (Figs. 1i, 1l and Supplementary Fig. 1e). However, an accumulation of PR+ cells accompanied by increased *Rankl* mRNA expression was observed (Fig. 1m and Supplementary Fig. 1g), indicating that luminal Rank loss might impair luminal progenitor expansion.

These observations were confirmed in MGs from K5^ΔRank^ mice with constitutive epithelial Rank deletion (Supplementary Fig. 1h). In this model, a high percentage of recombination was detected in luminal and basal lineages (Supplementary Fig. 1i), together with no changes in the luminal to basal ratio (Supplementary Fig. 1j). An increase in the number of PR+ cells was observed (Supplementary Fig. 1k), providing further support to the results obtained in K8^iΔRank^ mice.

Together, these data demonstrate that epithelial (basal or luminal) Rank signaling is not critical for fat pad invasion and that luminal Rank signaling is required for the maintenance of the luminal progenitor population during MG homeostasis.

### Luminal Rank loss impairs alveologenesis and leads to lactation failure

**Fig. 2:**
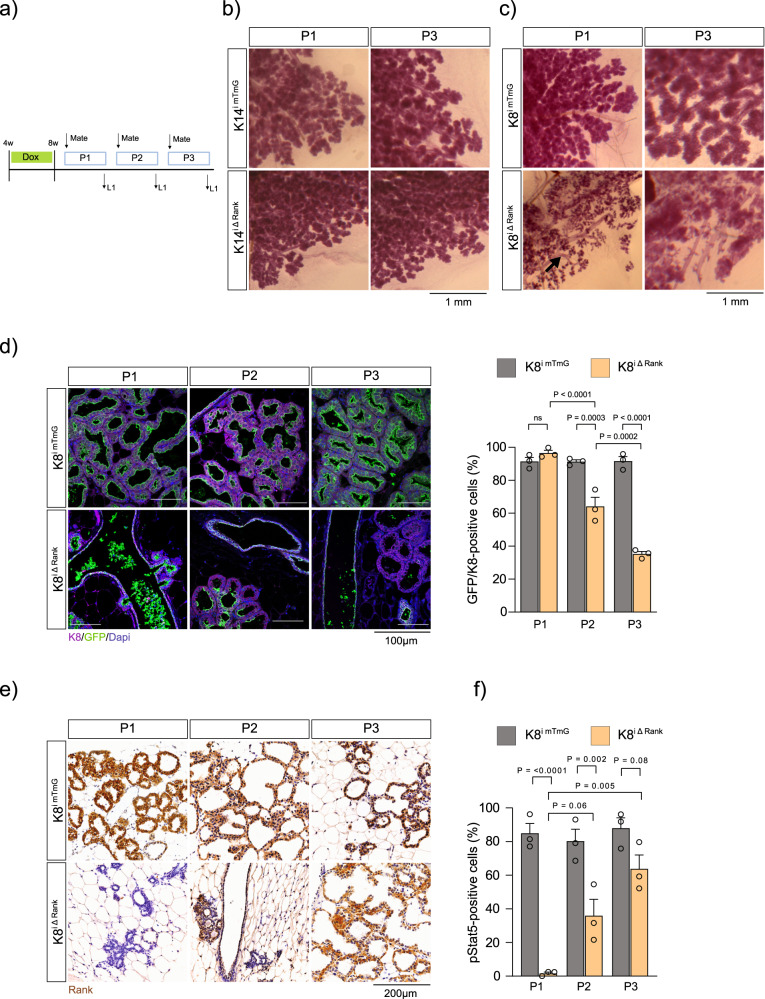
Luminal Rank loss results in lactation failure that is restored during successive pregnancies through the emergence of Rank-positive cells. **a** Experimental protocol used to establish the role of basal and luminal Rank during successive pregnancies in K14 and K8 transgenic models. **b** Whole mount MG staining from K14^imTmG^ and K14^iΔRank^ mice at L1 of pregnancy one (P1) and three (P3). **c** Whole mount MG staining from K8^imTmG^ and K8^iRank^ at L1 of P1 and P3. Black arrow indicates dilated ducts. **d** IF analysis of K8+ (magenta) and GFP+ (green) in MGs from K8^imTmG^ and K8^iΔRank^ mice, Dapi (blue) stains nuclei. Quantification of the number of GFP+ cells within the luminal (K8+) population at L1 from P1 (*p* = 0.98), P2 (*p* = 0.0003) and P3 (*p* < 0.0001) in K8^imTmG^ and K8^iΔRank^ (*n* = 3) is shown. **e** Rank immunohistochemistry at L1 from P1, P2 and P3 in K8^imTmG^ and K8^iΔRank^ mice. **f** Quantification of the percentage of pStat5+ cells in the luminal population at P1 (*p* < 0.0001), P2 (*p* = 0.002) and P3 (*p* = 0.08) at L1 in K8^imTmG^ and K8^iΔRank^ (*n* = 3). Data are represented as mean +/− SEM. Scale bars and significant *P* values are indicated in the graphs. *P*-values were calculated using Two-Way ANOVA with Tukey’s multiple comparisons (**d**, **f**) and were reported as the exact value when they were significant. Staining was quantified in 5 independent images from two tissue sections collected 100 µm apart (**d**, **f**). Source data are provided as a Source Data file. ns not significant.

Constitutive Rank deletion, either epithelial or ubiquitous, impairs lactation^2,5^; however, the contribution of Rank+ luminal/basal cells to this phenotype remains unexplored. To address the role of Rank signaling in each lineage during pregnancy, we analysed the ability of K14^iΔRank^ and K8^iΔRank^ mice to lactate (Fig. 2a).

Whole mount analysis at lactation day 1 (L1) of K14^iΔRank^ and K14^imTmG^ MGs failed to demonstrate any striking difference (Fig. 2b). Recombination at L1 was maintained at similar levels to that observed in the virgin MG (Supplementary Fig. 2a). Surprisingly, we detected several clones of GFP+ luminal cells within the L1 alveoli of both K14^iΔRank^ and K14^imTmG^ MGs (Supplementary Figs. 2a, b), an observation opposite to our findings in the virgin MG, in which no GFP+ cells were observed in the luminal population (Fig. 1c and Supplementary Fig. 1c). However, examination of lactating non-induced mice showed the existence of small clones of luminal GFP+ cells (in the absence of neighbouring basal cell recombination), indicating that these luminal clones represent K14 promoter leakiness during pregnancy (Supplementary Fig. 2c).

Rank signaling is a known regulator of stemness; we therefore explored the impact of basal Rank loss upon several cycles of pregnancy/involution. As observed at pregnancy one (P1), K14^iΔRank^ mice were able to lactate normally following a second (P2) and third pregnancy (P3) (Fig. 2b, Supplementary Figs. 2a, b). Lineage tracing of the basal GFP+ cells demonstrated that recombined cells remained stable throughout the pregnancies in both mouse lines (Supplementary Figs. 2a, b). This demonstrates that loss of basal Rank signaling does not impact basal cell expansion or functionality of the MG during pregnancy.

In contrast, K8^iΔRank^ females lost their litters at L1 of P1, indicating that Rank deletion exclusively in the luminal compartment led to lactation failure. K8^iΔRank^ MGs at this stage displayed significantly dilated ducts (black arrow, Fig. 2c), containing a whitish liquid and showing signs of defective differentiation. Indeed, K8^iΔRank^ MGs at L1 exhibited incomplete alveolar formation and reduced epithelial expansion, indicative of impaired alveologenesis (Supplementary Fig. 2d), which accounts for the mother’s inability to nurse the pups. Importantly, no differences in the serum levels of estradiol, progesterone, and prolactin at mid-pregnancy (G14.5) were found between genotypes demonstrating that K8^iΔRank^ and K8^imTmG^ mice received similar hormonal input during pregnancy (Supplementary Data 1).

To pinpoint the role of each hormone, we administered these hormones separately to virgin mice and observed reduced proliferation of K8^iΔRank^ luminal cells in response to progesterone and prolactin, while in response to estradiol proliferation was enhanced (Supplementary Fig. 2e). For this reason, we concluded that luminal Rank loss impairs the proliferative response of the mammary epithelia to progesterone and prolactin, leading to defective alveologenesis and subsequent lactation failure.

### Lactation is restored in K8^iΔRank^ mice upon subsequent pregnancies by the emergence of Rank-positive luminal cells

To our surprise, K8^iΔRank^ females were able to nurse a few pups after P2 and most of the litter by P3. Indeed, an almost normal lactating gland with proper developed alveoli was found at P3 (Fig. 2c, d). Quantification of GFP expression in K8^iΔRank^ and K8^imTmG^ MGs at P1 revealed a similar percentage of recombination in luminal cells in both models, comparable to that of virgins (Fig. 2d and Supplementary Fig. 3a). Analysis of non-induced K8^iΔRank^ MGs at P1 failed to demonstrate any evidence of promotor leakiness (Supplementary Fig. 3b). Interestingly, at P2 and P3, the percentage of GFP+ luminal cells decreased in K8^iΔRank^ while remaining constant in the control K8^imTmG^ mice (Fig. 2d and Supplementary Fig. 3a).

Rank/tdTomato immunostaining confirmed the presence Rank+/tdTomato+ luminal cells in K8^iΔRank^ MGs at P2/P3 (Fig. 2e and Supplementary Fig. 3c), indicating that the GFP dilution of the luminal cells was associated with the appearance of Rank+/tdTomato cells. Importantly, the key regulator of lactogenesis, pStat5, was absent in MGs of K8^iΔRank^ mice in P1 at L1, but its expression was progressively recovered in the lactating (L1) MGs at P2 and P3, in GFP+ and GFP− K8^iΔRank^ luminal cells, confirming a functional rescue (Fig. 2f and Supplementary Fig. 3d, e). The milk protein β-casein was detected not only in the lactating MGs of control K8^mTmG^ and K8^iΔRank^ mice at P2/P3, but also in ductal cells and aberrant alveoli of the dysfunctional K8^iΔRank^ at P1 (Supplementary Fig. 3f) demonstrating that luminal Rank-deficient cells were capable of producing some milk proteins, in line with the whitish liquid detected in the dilated ducts at L1.

Together our observations indicate that Rank-deficient luminal cells are progressively replaced by Rank+ cells to restore lactation, but only after the first pregnancy.

### Luminal Rank loss leads to aberrant alveolar differentiation in the virgin MG and impaired protein translation during pregnancy

**Fig. 3:**
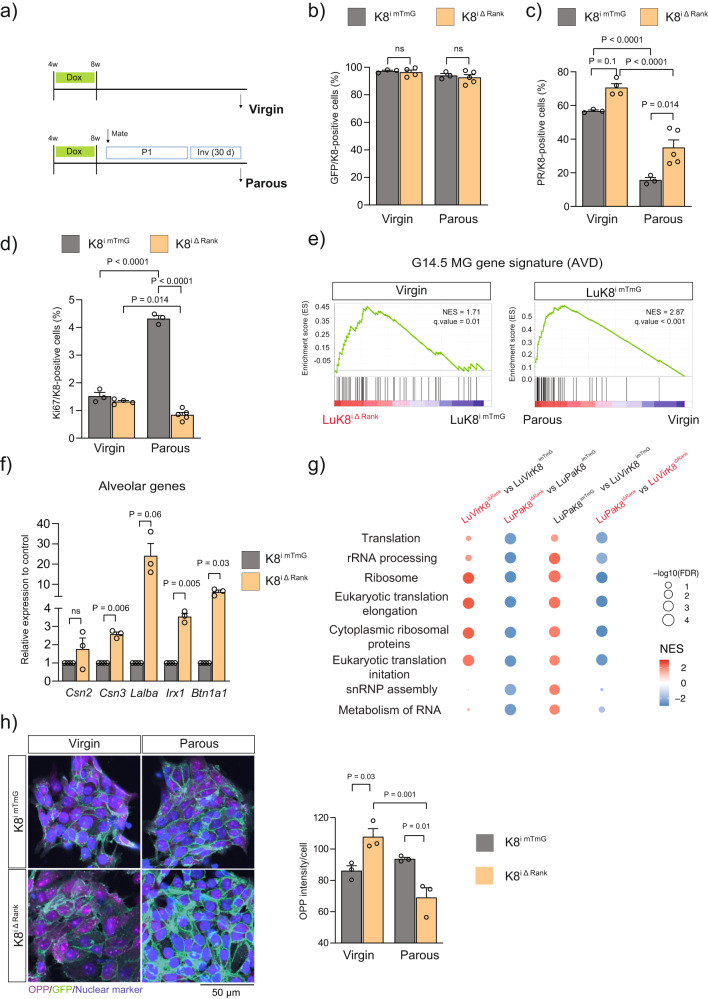
Rank deletion in luminal cells results in parity-associated protein translation defects. **a** Experimental protocol used to characterize the difference between virgin and parous K8^imTmG^ and K8^iΔRank^ females (for details see methods). **b** Quantification of GFP+ within K8+ cells in age-matched virgin (*n* = 3 K8^imTmG^; *n* = 4 K8^iΔRank^; *p* = 0.98) and parous MGs (*n* = 3 K8^imTmG^; *n* = 5 K8^iΔRank^; *p* = 0.93). **c** Percentage of luminal differentiated cells (PR+) in the K8+ lineage in virgin (*n* = 3 K8^imTmG^; *n* = 4 K8^iΔRank^; *p* = 0.11) and parous stage (*n* = 3 K8^imTmG^; *n* = 5 K8^iΔRank^; *p* = 0.01). **d** Quantification of proliferation (Ki-67) in luminal (K8+) cells in virgin (*n* = 3 K8^imTmG^; *n* = 4 K8^iΔRank^; *p* = 0.67) and parous (*n* = 3 K8^imTmG^; *n* = 5 K8^iΔRank^; *p* < 0.0001) MGs. **e** GSEA profiles of alveolar progenitor differential gene set obtained at G14.5 MG (AVD) between virgin and parous luminal (Lu) cells of the indicated genotypes. **f** qPCR analysis of differentially expressed lactation genes (*Csn2* (*p* = 0.33)*, Csn3* (*p* = 0.006)*, Lalba* (*p* = 0.06)*, Irx1* (*p* = 0.005)*, Btn1a1* (*p* = 0.03)) between virgin K8^imTmG^ (*n* = 4) and K8^iΔRank^ (*n* = 3) mice in sorted luminal GFP+ cells. **g** Bubble plot representation showing selected significant gene sets related to ribosomal/translation in the different biological settings indicated from luminal cells of K8^imTmG^ and K8^iΔRank^ mice. **h** Representative images of protein synthesis signal (OPP signal in magenta) in primary luminal cells (GFP+ in green) and quantification of protein synthesis (average intensity of OPP signal per cell/per field) from virgin (*p* = 0.03) and parous (*p* = 0.01) K8^imTmG^ and K8^iΔRank^ mice (*n* = 3). Data are represented as mean +/− SEM. Scale bars and significant *P* values are indicated in the graphs. *P*-values were calculated by Two-Way ANOVA with Tukey’s multiple comparisons (**b**, **c**, **d**, **h**) and One Sample *T*-Test (**f**). Staining was quantified in 5 independent images from two tissue sections collected 100 µm apart (**b**, **c**, **d**). Staining was quantified in 5 independent images (**h**). Source data are provided as a Source Data file. ns not significant.

Pregnancy causes a series of molecular changes in MECs that influence the activation of pregnancy-related programs during the re-exposure to pregnancy hormones^12,13^. Thus, we sought to identify the changes that pregnancy and involution impose on K8^iΔRank^ MGs that may underlie their distinct functionality between P1 and P2. Age matched virgin and parous (30 days post-involution) MGs were analyzed for GFP expression, luminal differentiation and proliferation (Fig. 3a). The percentage of GFP+ cells within the luminal population remained constant in virgin and parous MGs ruling out involution as a mechanism for the loss of GFP+/Rank− cells (Fig. 3b).

Parity promotes a reduction in the number of PR+ cells^14^. Interestingly, the increase in the relative frequency of PR+ cells previously observed in K8^iΔRank^ compared to K8^imTmG^ virgin females is still present in parous glands, demonstrating the importance of Rank signaling in the maintenance of the luminal progenitor pool (Fig. 3c). Proliferation levels (Ki67+ cells) were similarly low in K8^imTmG^ and K8^iΔRank^ virgin MGs, while after parity, luminal proliferation was increased in the control K8^imTmG^ but not in the K8^iΔRank^ MGs (Fig. 3d).

To address the molecular mechanisms underlying the distinct behavior of virgin and parous K8^iΔRank^ MECs during pregnancy and lactation, we isolated and performed a transcriptomic analysis in basal and luminal cells from K8^imTmG^ and K8^iΔRank^ age matched virgin and parous MGs (Supplementary Fig. 4a; Supplementary Data 2). Sample identity was confirmed by heatmap analysis of known luminal and basal markers, while expression of *Csn2* was used to discriminate between virgin and parous stages (Supplementary Fig. 4b).

We first interrogated our gene data set for known MG lineage differentiation gene sets^11,15^. Strikingly, virgin luminal K8^iΔRank^ cells showed a significant enrichment in gene signatures obtained from G14.5 MGs identifying alveolar cells (Fig. 3e, Supplementary Data 3)^11^. This gene set was also upregulated in control parous luminal cells compared with their virgin counterparts (Fig. 3e). The aberrant expression of these alveolar genes was confirmed by qPCR on sorted virgin luminal cells from K8^iΔRank^ MGs (Fig. 3f). These data suggest that loss of Rank signaling in virgin luminal cells induce an alveolar-like differentiation status, reminiscent of pregnancy. Interestingly, the alveolar gene signature is downregulated in the luminal parous population from K8^iΔRank^ mice compared to their control counterparts (parous K8^mTmG^), although no differences were observed when compared to the virgin K8^iΔRank^ luminal cells (Supplementary Fig. 4c).

Then, we aimed at understanding the consequences of parity in K8^iΔRank^ luminal cells and corresponding controls. Enrichment of gene sets related to cell cycle/proliferation was observed upon parity in control but not in K8^iΔRank^ MECs (Supplementary Fig. 4d; Supplementary Data 4). Indeed, a reduction in cell cycle/proliferation gene sets was seen in Rank-depleted parous compared to control counterparts confirming our previous data (Fig. 3d, Supplementary Fig. 4d). Examination of the top pathways found by GSEA throughout our experimental settings revealed multiple gene sets related to protein translation: in the virgin MGs, loss of luminal Rank signaling results in an enrichment of protein synthesis pathways (Fig. 3g; Supplementary Data 4), in line with their aberrant secretory alveolar differentiation (Fig. 3e). Strikingly, a strong downregulation of these same protein synthesis pathways was found in the parous luminal Rank-depleted context relative to the parous control and virgin Rank-depleted counterparts (Fig. 3g). As an example, the ribosome gene set showed a mirror image between the virgin and parous context in K8^iΔRank^ luminal cells (Supplementary Fig. 4e). We thus analysed whether protein translation was affected in K8^iΔRank^ luminal cells using an in vitro assay based on puromycin (fluorescently labelled, named OPP) incorporation into nascent proteins. OPP incorporation was significantly increased in virgin K8^iΔRank^ luminal cells compared to virgin controls (Fig. 3h), indicating enhanced protein synthesis. In contrast, luminal Rank deletion resulted in a reduction in protein synthesis in parous MGs (Fig. 3h), confirming the transcriptomic observations.

Overall, these findings suggest that luminal Rank deletion leads to an aberrant alveolar-like differentiation with a high translational status in the virgin stage. Therefore, we propose that the aberrant differentiation in Rank-deficient luminal cells hampers the development of functional alveoli during the first pregnancy. During the subsequent pregnancies, the presence of these dysfunctional cells with impaired translation triggers the rescue by Rank-expressing cells to restore lactation.

### Loss of luminal Rank results in parity-induced basal bipotency through enhanced NF-κB activation

Luminal cells have been demonstrated to restrain basal cell bipotency^16^. Indeed, the bipotency of embryonic basal cells can be awakened in adult mice when there is a disruption in luminal-basal cell communication, as demonstrated by transplantation assays or luminal cell ablation^16–20^. Our findings suggest that the loss of luminal Rank has a detrimental effect on the fitness of parous luminal cells that might trigger bipotency of the Rank+ basal cells to restore lactation.

To investigate this hypothesis we subjected K5^ΔRank^ females, in which Rank is deleted in both basal and luminal cells (Supplementary Fig. 1h, i), to different rounds of pregnancy. Lactation was not restored in successive pregnancies (Supplementary Fig. 5a), despite the presence of 10% of unrecombined luminal cells (Supplementary Fig. 1i), indicating that GFP− Rank+ luminal cells observed at P2 and P3 in K8^iΔRank^ MGs are derived from the basal epithelium rather than the few unrecombined luminal cells (Fig. 1j and Supplementary Fig. 1i). Furthermore, the lack of lactation rescue in the K5^ΔRank^ MGs raises the possibility that basal Rank signaling might be involved in basal to luminal conversion in the K8^ΔiRank^ context.

**Fig. 4:**
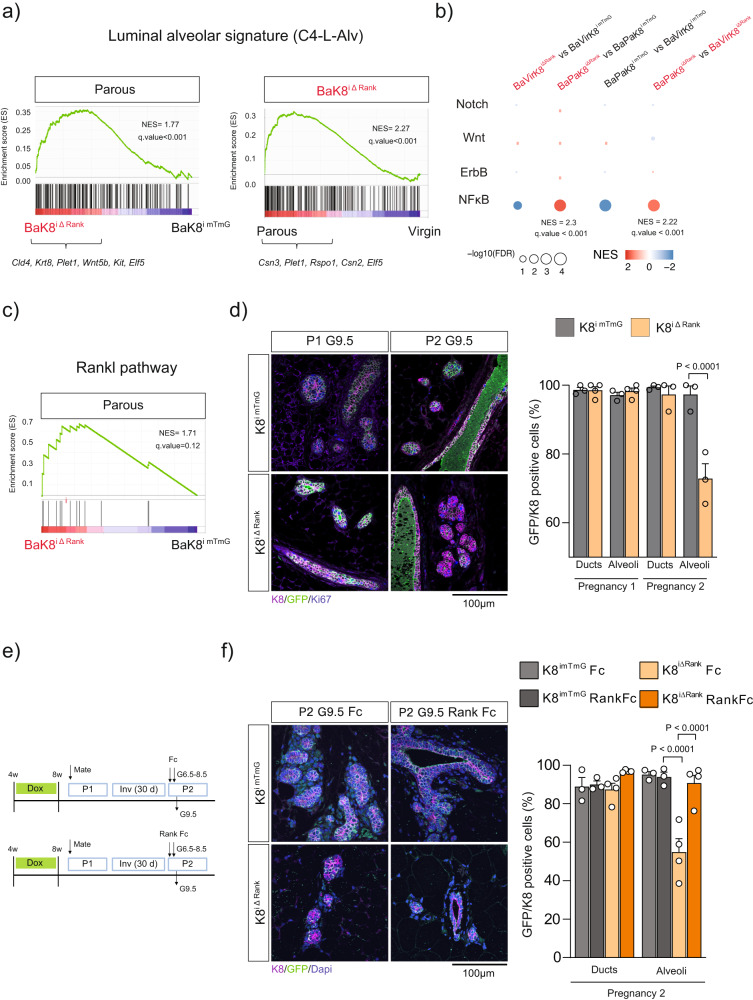
Rank deletion in luminal cells leads to parity-induced basal bipotency in developing alveoli through Rank pathway activation in basal parous cells. **a** GSEA profile of luminal alveolar identity gene set (C4-L-Alv) between virgin and parous Rank+ basal (Ba) cells of the indicated genotypes. **b** Bubble plot representation showing selected GSEA related to signaling pathways involved in basal bipotency in basal cells in the different biological settings indicated. **c** GSEA profile of Rankl pathway between Rank+ parous basal (Ba) cells of the indicated genotypes. **d** IF analysis of K8 (magenta), GFP (green) and Ki67 (blue) at P1 and P2 G9.5 from K8^imTmG^ and K8^iΔRank^ MGs. Quantification of recombination (K8+/GFP+) in ducts (*p* > 0.99) and alveoli (*p* > 0.99) at G9.5 from P1 (*n* = 3 K8^imTmG^; *n* = 4 K8^iΔRank^); and P2 ducts (*p* = 0.92) and alveoli (*p* < 0.0001) (*n* = 3). **e** Experimental protocol used to inhibit Rank signaling in basal cells of K8^iΔRank^ mice at early P2. During P2 , both control and K8^iΔRank^ mice were treated intraperitoneally with Rank-Fc and control Fc at G6.5 and G8.5, and sacrificed at G9.5. **f** IF analysis of K8 (magenta), GFP (green), and Dapi (blue) at P2 G9.5 from K8^imTmG^ and K8^iΔRank^ mice treated with Fc and Rank-Fc. Quantification of recombination (K8 + /GFP + ) in ducts (*p* > 0.99) and alveoli (*p* < 0.0001) at G9.5 P2 (*n* = 3 K8^imTmG^; *n* = 4 K8^iΔRank^) after Fc treatment and ducts (*p* > 0.83) and alveoli (*p* > 0.99) at G9.5 P2 after Rank Fc treatment (*n* = 3 K8^imTmG^; *n* = 4 K8^iΔRank^). Data are represented as mean +/− SEM. Scale bars and significant *P* values are indicated in the graphs. *P*-values were calculated by ANOVA with Tukey’s multiple comparisons (**d**, **f**). Staining was quantified in 5 independent images from two tissue sections collected 100 µm apart (**d**, **f**). Source data are provided as a Source Data file. ns not significant.

Aiming to understand putative changes in parous basal cells which may favor basal to luminal transition in the luminal Rank loss scenario, we interrogated our transcriptomic data of paired virgin and parous basal cells (with intact Rank) sorted from K8^iΔRank^ and control MGs (Supplementary Figs. 4a, b, Supplementary Data 2 and 4). Comparison with lineage specific gene signatures derived from the integration of five scRNA-seq datasets^21^ (Supplementary Data 3) revealed strong enrichment of a luminal alveolar signature in basal parous cells from K8^iΔRank^ mice compared to their virgin counterparts and parous controls K8^imTmG^, but not in the other two comparisons (Fig. 4a, Supplementary Fig. 5b). These results indicate that parity upholds basal cell differentiation towards a luminal alveolar lineage in K8^iΔRank^ MGs. To identify the signaling pathways involved in basal to luminal differentiation in parous glands, we analysed pathways previously demonstrated to be involved in basal bipotency^16^. NF-κB pathway was the only pathway upregulated in parous basal cells from K8^iΔRank^ MGs (in which bipotency is observed) compared to the virgins and parous control counterparts (Fig. 4b). As expected, basal cells isolated from control MGs gave rise to both luminal (K8+) and basal (K5+) compartments in organoid cultures. However, in the presence of NF-κB inhibitors, smaller organoids and a reduction in K8 expression were observed (Supplementary Fig. 5c), indicating that activation of NF-κB signaling pathway is critical for basal to luminal transition.

### Inhibition of Rank signaling impairs basal bipotency in the developing alveoli of parous glands

NF-κB is the main downstream mediator of Rank signaling and our transcriptomic analysis revealed that Rankl signaling increases in basal parous cells of K8^iΔRank^ mice (Fig. 4c; FDR < 0.15). We reasoned that luminal Rank depletion increases the availability of Rankl (produced by hormone-sensing luminal cells) for Rank+ basal cells in K8^iΔRank^ mice, enhancing basal Rank pathway activation, which may contribute to awake basal bipotency during the second pregnancy. To determine the onset of Rank+ luminal cells appearance and pinpoint the underlying molecular mechanism, we compared P1 and P2 MGs at the initiation of alveologenesis, gestation day 9.5 (G9.5) (Supplementary Fig. 5d). Full luminal recombination was found in ducts and alveoli from control and K8^iΔRank^ luminal cells at P1 G9.5 (Fig. 4d) and higher levels of proliferation were observed in the alveoli compared to the ducts irrespectively of luminal Rank expression in P1 (Supplementary Fig. 5e). However, at P2 G9.5, GFP dilution occurred exclusively in the developing alveoli and not in the ducts from K8^iΔRank^ MGs (Fig. 4d), despite impaired proliferation in K8^iΔRank^ ducts and alveoli (Supplementary Fig. 5e). The Rank+ luminal population (GFP−) that emerges at K8^iΔRank^ MGs at P2 showed proliferation levels comparable to control luminal cells, explaining the progressive dilution of the less proliferative luminal Rank-depleted cells (GFP+/Ki67+ at P2) (Supplementary Fig. 5f) during successive pregnancies. Rank deletion did not promote apoptosis (determined by cleaved caspase 3 staining) in MGs, neither at P1 nor P2 in G9.5 (Supplementary Fig. 5g), ruling out that the replacement of Rank-deficient luminal cells is driven by luminal cell death.

**Fig. 5:**
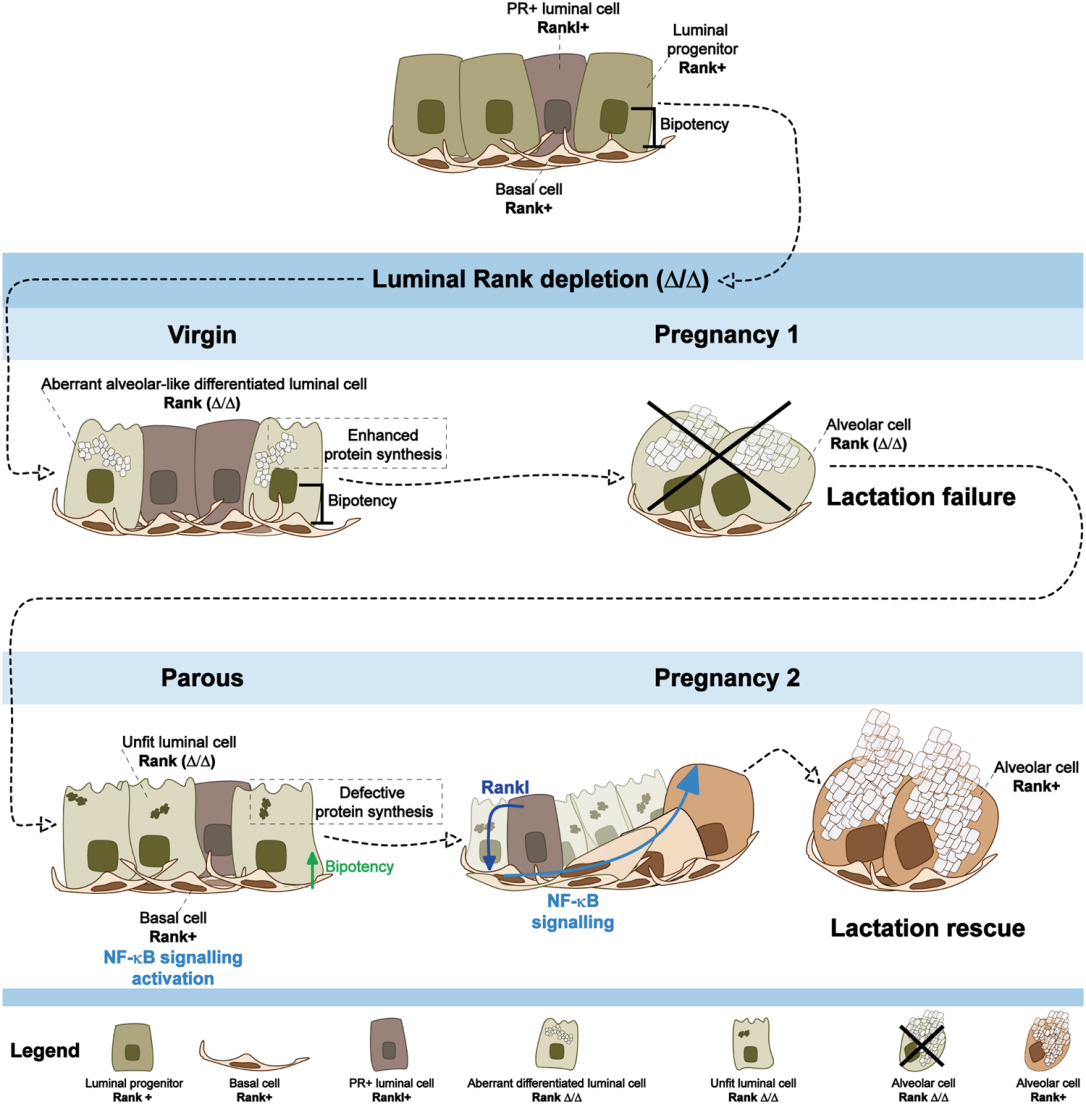
Proposed model detailing the phenotype of luminal Rank depleted mice and the underlying molecular mechanisms. In virgin MGs, luminal Rank loss promotes an aberrant alveolar differentiation with enhanced protein synthesis and expansion of the hormone-sensing cells (PR+) that produce Rankl and lead to lactation failure. Rank-deleted parous luminal cells show a reduction in protein synthesis ability. Upon the following pregnancy, these unfit luminal cells lacking Rank cannot cope with the high translational demands required for lactation. This, together with the increased availability of Rankl for basal cells during early pregnancy, enhances Rank/NF-κB signaling in the basal cell population and activates basal bipotency in developing alveoli. Basal to luminal differentiation results in the replacement of Rank-depleted with Rank-proficient luminal cells in the alveoli to restore lactation.

At this early gestation stage, we confirmed the presence of Rankl in luminal cells from both genotypes, with higher levels observed in K8^iΔRank^ mice (Supplementary Fig. 5h), denoting a possible contribution of luminal Rankl in the activation of basal bipotency. To address this point, we inhibited pharmacologically Rankl (by injecting Rank-Fc and the corresponding Fc control) in K8^iΔRank^ and K8^imTmG^ mice during early P2 (G6.5 and G8.5) (Fig. 4e). Rankl inhibition restricted the basal to luminal conversion within alveoli, denoted by a recovery of the frequency of luminal GFP+ cells in Rank-Fc-treated mice, similar to the levels seen in K8^imTmG^ alveoli (Fig. 4f). These results support that the activation of basal Rank signaling is required for basal to luminal transition at P2. In consequence, we propose that loss of luminal Rank in the parous MGs enhances Rank/NF-κB signaling in the basal population and prompts basal bipotency in the “unfit” alveoli to restore lactation (Fig. 5).

## Discussion

MG terminal differentiation is accomplished through a series of complex steps involving tissue remodeling, expansion of the epithelial ductal tree, and differentiation into alveolar structures culminating in milk production. It has been demonstrated that parity permanently changes MG physiology; epigenomic, transcriptional, and metabolic changes have been found in the mammary epithelium, and more recently, changes in the stromal environment have been also documented^13,22^.

Rank signaling has been demonstrated to be a key player in MG development^2,6^, however, its precise function in the different MG epithelial lineages has not been accurately addressed, as previously characterized Rank mouse models were either constitutive knockouts or transgenic lines expressing Rank under the MMTV promoter^2,10^. We have therefore explored the putative role of Rank in basal and luminal cells during fat pad invasion, pregnancy, and lactation, as well as in the tissue renewal potential over successive pregnancies. Consistent with previous data on pubertal MG^2,6^, Rank deletion in either basal or luminal MECs did not affect fat pad invasion, nor did it induce major histological changes. However, loss of luminal Rank signaling led to a reduction in the luminal progenitor population in favor of hormone-sensing cells in the virgin MG, accompanied by the inability of Rank-depleted luminal cells to respond to progesterone and prolactin mitogenic cues.

The critical role of Rank in progesterone signaling has been extensively demonstrated during pregnancy^4–6,10^ and in the development and progression of progesterone-driven mammary cancer^3,23^. Surprisingly, transcriptomic analysis of Rank-deleted luminal cells revealed a precocious differentiation towards an alveolar progenitor-like identity, with aberrant expression of some lactation genes generally expressed in pregnancy, in the absence of changes in pregnancy-related hormones. Together, these results demonstrate that Rank signaling, in the virgin MG, represses a transcriptional program controlling early mammary alveolar differentiation while promoting progesterone-driven proliferation during the oestrous cycle. Our data uncovers a role for luminal Rank signaling in MG homeostasis independent of progesterone-induced mitogenesis.

We then asked whether the impaired lactation phenotype previously described for constitutive epithelial Rank loss^2,5^ stemmed from Rank signaling in the luminal, basal, or both populations. Phenotypic and lineage tracing analysis indicated no impact of basal Rank loss on alveologenesis/lactation, nor in long-term renewal of this population upon multiple pregnancies. In contrast, luminal Rank deletion resulted in lactation failure, resembling that of ubiquitous and epithelial Rank-null mouse models. To our surprise, lactation was progressively recovered in successive pregnancies. Lineage tracing analysis demonstrated the replacement of luminal Rank-depleted (GFP+) by Rank+ (Tom+) cells within the luminal layer, a conversion starting early at second pregnancy in the developing alveoli, structures responsible for milk production in which Rank is strongly expressed^10^. GSEA in sorted parous basal cells from luminal Rank-depleted mice evidenced the acquisition of “luminal alveolar-like” features that, together with the inability of Rank-deficient basal cells to recover lactation (as seen in the K5^ΔRank^ epithelial mouse model), allowed us to identify the basal lineage as a putative source of GFP−/Rank+ luminal cells. We thus propose that the combination of Rank deletion and the pregnancy/lactation/involution cycles impinges molecular changes that hamper luminal cell expansion and promote basal cell bipotency in the following pregnancies. Indeed, Centonze et al. demonstrated that luminal cell apoptosis, even at low levels, activates a homeostasis mechanism involving the induction of basal cell bipotency and basal to luminal conversion to restore MG functionality^16^. In our models, we did not detect major changes in apoptosis upon Rank deletion. Instead, transcriptomic and phenotypic analysis demonstrated that luminal parous Rank-depleted cells show impaired protein translation and proliferative capacity that might result in an inability to cope with the high translational demands of successive pregnancy/lactation cycles and, hence, long-term maintenance. Although parous ductal and alveolar cells showed defective proliferation, replacement of the Rank null luminal cells occurred only within the alveoli, where Rank activation is essential^10^ and milk production requires intensified protein synthesis.

Transcriptomic analysis of the basal population in our luminal Rank null model showed that the luminal-like transition is accompanied by an upregulation of the NF-κB pathway. Using an NF-κB reporter mouse model, it has been previously demonstrated that luminal progenitors show activated NF-κB unlike basal/MaSCs^24^, suggestive of a possible involvement of this pathway in luminal identity. Furthermore, van Weele and co-authors have shown that TNFα treatment augments the clonogenic properties of basal cells, resulting in an increased number and size of organoids^25^. Our own data, using NF-κB inhibitors supports the conclusion that NF-κB signaling favors basal cell stemness and expansion. This observation is in line with findings from Brugge´s group showing that aging myoepithelial cells lose differentiation markers such as K5 and α-Sma, while displaying increased NF-κB signaling^26^.

Finally, we uncovered that Rank signaling in the basal population is required for bipotency. Rankl pharmacologically inhibition in our luminal Rank model prevented GFP dilution in the developing alveoli and the rescue of the lactation phenotype.

In conclusion, we demonstrate that Rank deletion in luminal cells has pleiotropic effects beyond alveologenesis and lactation. Loss of luminal Rank changes the mammary gland hierarchy, resulting in the accumulation of PR+/Rankl-producing cells that differentiate into alveolar-like progenitors. Under the action of the cues from pregnancy hormones, these aberrantly differentiated cells are unable to proliferate properly and fully differentiate into alveolar cells. Following involution these cells feature an impaired translational ability indicative of a tissue damage response. This hint, combined with an excess of available Rankl, favours NF-κB activity in the basal cells, resulting in bipotency awakening and restoration of the mammary epithelium and function.

## Methods

### Animal models

The research involving animals was conducted at the IDIBELL and CNIO animal facilities, adhering to protocols approved by the respective Committee on Animal Care and in accordance with both national and European Union regulations. Specifically, all animal experiments were approved by our Institutional Animal Care and Use Committee (IACUC) and by the Ethical Committee for Animal Experimentation (CEIyBA) (PROEX028/19 and 161.2/21). Mice were maintained in cages (specific pathogen free, SPF) under controlled conditions of humidity (55 ± 5%), temperature (21 ± 1 °C), cycles of light/dark of 12/12 h, and with food and water were given ad libitum.

Rank flox/flox (Rank^fl/fl^) were provided by Dr. Joseph Penninger^27^ and crossed with either K14rtta:Tet-O-Cre or K8rtta:Tet-O-Cre, kindly provided by Cedric Blanpain, or K5-Cre^28^. The resulting models were crossed with the reporter mouse line Rosa^mT/mG^ (MGI 3716464) to perform lineage tracing analysis. The following experimental genotypes were used in the current study: K8^iΔRank^ (K8rtta: TET-O-CRE: Rank^fl/fl^: Rosa^mT/mG^); K8^imTmG^ (K8rtta:TET-O-CRE: Rosa^mT/mG^); K14^iΔRank^ (K14rtta:TET-O-CRE: Rank^fl/fl^: Rosa^mT/mG^; K14^imTmG^ (K14rtta:TET-O-CRE: Rosa^mT/mG^); K5^ΔRank^ (K5Cre: Rank^fl/fl^: Rosa^mT/mG^) and K5^mTmG^ (K5-Cre: Rosa ^mT/mG^). The mice used in this study were females and were maintained in a mixed background.

Cre recombination was achieved in the inducible mouse models by administering doxycycline in the drinking water with sucrose (1 mg/ml dox in 20 mg/ml sucrose, changed every 2–3 days), starting at week 4 of age during 4 consecutive weeks. In the pregnancy experiments, once the mice reached adulthood, they were mated and allowed to undergo pregnancy and lactation. On lactation day 10, the pups were removed, and the mice were allowed to undergo involution for a period of 30 days. After the involution period, the mice were subsequently mated to undergo two more pregnancies if necessary. In the case of K8^iΔRank^ mice and K5^ΔRank^ mice, which exhibited lactation failure at lactation day 1, the mice were mated again after 30 days to simulate a normal involution process.

### In vivo treatments

For estradiol treatment, mice received drinking water with 17β-estradiol at 8 μg/ml (Sigma) and were sacrificed 3 weeks after the first treatment. For progesterone treatment, subcutaneous implantation of 90-day release 50 mg medroxyprogesterone acetate (MPA) pellets (Innovative Research of America) was performed, and the mice were sacrificed 3 weeks post-implantation. To administer prolactin treatment, mice were given intraperitoneal injections of prolactin (1 μg/g animal) twice a day for 5 consecutive days and mice were sacrificed at day 5. For Rankl pharmacological inhibition during the early second pregnancy, females were injected intraperitoneally with PBS-diluted Fc and Rank-Fc (Amgen Inc, Thousand Oaks, CA, www.amgen.com) at a dosage of 10 mg/kg at gestation days G6.5 and G8.5. The animals were subsequently sacrificed at G9.5.

### Sexual hormonal quantification in serum

For the analysis of steroid hormones in serum, an HPLC analysis was conducted^29^. The serum sample was first centrifuged at 20,200g for 10 min and filtered through a 0.2 μm nylon filter. Then, 170 μL of the serum was processed in the SPE system through various elution steps to retain the steroid hormones. Subsequently, chromatography was performed using a C18 column at 35 °C with a flow rate of 300 μL/min (particle size: 2.6 μm, length: 10 cm, diameter: 3 mm), and MS/MS detection was employed to monitor each target steroid. Prolactin levels in serum were measured using an ELISA kit (Life Technologies) following the manufacturer’s instructions.

### Whole mount

Briefly, inguinal MGs were excised and stretched onto a glass slide. For virgin MGs, endogenous GFP signal was visualized and captured using a Leica MZ16F vertical fluorescence stereomicroscope. Due to the high auto-fluorescence at L1 stage, standard Carmine aluminium staining was performed.

### Immunofluorescence (IF) and immunohistochemistry (IHC)

Mouse tissue samples were fixed in formalin overnight at 4 °C and embedded in paraffin. Five µm sections were cut for histological analysis and processed for IF or IHC analysis after antigen heat retrieval as detailed in^8,9^. For IF, tissue slides were incubated overnight (4 °C) with the primary antibodies: K8 (Developmental Studies Hybridoma Bank; 1/400), K14 (AF-64, Covance, 1/400), Ki67 (SP6, 16667 Abcam, 1/500), PR (SP2, 12683667, Fisher, 1/200), GFP (ab13970, Abcam, 1/500), pStat5 (9359 S, Cell Signaling, 1/200), β-casein (sc-166530, Santa Cruz, 1/200). Slides were then incubated with corresponding fluorochrome-conjugated secondary antibodies (Jackson Immunoresearch, 1/400) and DAPI (Sigma) to stain cell nuclei and then mounted with Prolong Gold Antifade reagent (ThermoFisher Scientific). Confocal analysis was carried out using a Leica 7100 confocal microscope. For IHC, primary antibodies were incubated overnight at 4 °C (cleaved Caspase 3 (Cell Signaling, 1/100), Rankl (R&D, AF462, 1/100), Rank (R&D, AF692, 1/200), tdTomato (Rockland, 600-401-379, 1/100)), detected with biotinylated secondary antibodies and streptavidin horseradish peroxidase (Vector), and revealed with DAB substrate (DAKO). For Rankl IHC, heat antigen retrieval (citrate buffer pH 6.0) was done for 10 min followed by 2 min at maximum pressure and a cool down of 25–30 min. Slides were then treated with Pronase 100 μg/mL for 10 min before incubation with Rankl antibody. For Rank IHC antigen retrieval was achieved by digestion with Protease TypeXXIV (5 units/ml) (Sigma, P8038) for 5 min at room temperature^10^. Positive staining was quantified in 5 independent images at 20x magnification (OLYMPUS AX70) from 2 sections collected 100 µm apart. Validation of the antibodies is available in the website of each company.

### Single cell isolation for flow cytometry sorting

MGs were dissected mechanically using a McIlwain tissue chopper. Prior to digestion, lymph nodes were removed, and the glands were enzymatically digested using a digestion medium composed of Dulbecco’s modified Eagle’s medium (DMEM)/F-12, 0.3% collagenase A, 2.5 U/mL dispase, 20 mM HEPES, and 1% penicillin–streptomycin for 40 min at 37 °C. After each step, samples were washed with Leibowitz L15 medium containing 10% fetal bovine serum (FBS). To eliminate erythrocytes, the samples were treated with a hypotonic lysis buffer (Lonza Iberica). Single cells were isolated through sequential treatments, beginning with trypsin (PAA Laboratories) for 2 min at 37 °C, followed by dispase/DNAse at 37 °C for 10 min. Cell aggregates were removed by filtering the cell suspension through a 40 μm strainer. MECs were then counted, resuspended in HBSS with 2% FBS and 2 mM EDTA, and blocked with FcR blocking reagent (Miltenyi Biotec) for 10 min on ice. The cells were incubated for 45 min on ice with the corresponding surface antibodies: CD24-alexa 700 (1.25 µg/mL, HMb1-1, BD Pharmingen), CD49f-alexa 647 (2.5 µg/mL, GoH3, BD Pharmingen). Flow cytometry was used to exclude lymphocytes and endothelial cells, employing CD45-PECy7 (0.125 µg/mL, 30-F11 Biolegend) and CD31-PECy7 (0.5 µg/mL, 390 Biolegend) antibodies, respectively. GFP and tdTomato fluorescence were analyzed based on the intrinsic fluorescence of mammary epithelial cells. Cell sorting was conducted using MoFlo (Beckman Coulter) with a 100 mm tip at 25 psi.

### RNA isolation, RT-PCR, and gene expression analysis

Total RNA from sorted MECs was extracted using the RNAeasy Micro kit (Qiagen) and reverse-transcribed with NZY Reverse Transcriptase kit (NZYtech MB124) according to the manufacturer’s instructions. qPCRs were performed with LightCycler® 480 SYBR green; primer sequences are indicated in Supplementary Data 5. Ct analysis was performed using LightCycler 480 software (Roche).

### RNA sequencing

Cells were directly collected from the sorter into the lysis buffer from RNeasy micro kit (Qiagen). Samples were mechanically homogenized and RNA was extracted according to the manufacturer´s instructions. From total RNA samples, 500 ng with an average RIN = 6.9 (range: 2.5–8.6) were processed into cDNA sequencing libraries with the “QuantSeq 3’mRNA-Seq Library Prep Kit (FWD) for Illumina” (Lexogen, Cat.No. 015) with a UMI Second Strand Synthesis module. Briefly, library generation is initiated by reverse transcription with oligodT priming, followed by a random-primed second strand synthesis. While primers from both steps contain Illumina-compatible sequences, random primers additionally feature 6 nt long Unique Molecular Identifier (UMI) tags. Libraries are completed by PCR and sequenced on an Illumina NextSeq 550 (with v2.5 reagent kits) by following the manufacturer’s protocols. This library preparation kit generates directional libraries stranded in the sense orientation, i.e., the read1 (the only read in single read format) has the sense orientation. Eighty-six-base-pair single-end reads were pre-processed with BBDuk (https://jgi.doe.gov/data-and-tools/software-tools/bbtools/bb-tools-user-guide/bbduk-guide/) to remove possible traces of rRNA, adapters and polyA tails, as recommended by Lexogen. UMI-tools^30^ were used to extract UMIs from sequenced reads, which were then aligned to the mouse genome (GRCm39) with Bowtie2^31^. SAMtools^32^ was used to create the corresponding sorted BAM files. UMI-tools were used again for final read deduplication, and the resulting BAM files were converted to FASTQ^33^ format with BEDtools^34^. FASTQ files were analysed with the nextpresso pipeline^35^. Sequencing quality was checked with FastQC v0.11.9 (https://www.bioinformatics.babraham.ac.uk/projects/fastqc/). Differential expression and normalization were performed with DESeq2^36^, keeping only those genes where the normalized count value was higher than 10 in at least 25% of the samples (the Gencode vM26 gene annotation for GRCm39 was used). Finally, those genes that had an adjusted p-value below 0.05 FDR were selected. GSEAPreRanked^37^ was used to perform gene set enrichment analysis for the selected gene signatures on a pre-Ranked gene list, setting 1000 gene set permutations. Only those gene sets with significant enrichment levels (FDR *q*-value < 0.25) were considered. Bubble plots were built with ggseabubble (https://gitlab.com/bu_cnio/ggseabubble). GSEA was performed with the gene signatures identified by scRNASeq in^11^ and^21^.

### OPP experiments

Single cells were obtained by standard tissue digestion (see above flow cytometry sorting protocol) and epithelial cells were obtained by positive selection using mouse EPCAM beads magnetic beads (mouse CD326 (EpCAM) MicroBeads from Mylteny Biotec) according to the manufacturers’ instructions. Briefly, 100,000 cells were platted per well (8 well culture chamber plate, Indibi) in DMEM/F12 with 10% FBS, 1% ITS, and 1% penicillin–streptomycin for 24 h Cells were then incubated with OPP (ClickIt OPP Alexa 647) for 1 h following the kit protocol. OPP signal was measured within the GFP+ luminal cells and IF images were acquired in a Leica 7100 confocal microscope. Signal intensity was quantified using the macro developed by Joan Ripolles (IDIBELL Bioimaging platform).

### NF-κB assay

For NF-κB inhibition assays, 5,000 sorted basal MECs from WT virgin MGs were plated in Matrigel 3D cultures in basal medium (DMEM F12, 1% FBS, 1% Penicilin/Streptomicyn, 1X B27, 10 ng/mL EGF, 5 μg/mL insulin, 100 ng/mL cholera toxin and 10 μM Y-27632 (ROCK inhibitor, Sigma)). NF-κB inhibitors were added 24 h later (5 µM BAY65 (Calbiochem) or 18 μM SN50 (Enzo)) and cells were cultured for 6 days. Rankl (1 μg/ml) was added 2 h after NF-κB inhibitors. The treatment was refreshed daily for BAY or every 48 h for SN50 inhibitors to avoid their degradation. Upon medium removal, 3D acinar structures were stained^38^. Briefly, acini were fixed in 2% paraformaldehyde (20 min), permeabilized with PBS containing 2% Triton X-100 (30 min), and washed with PBS-Glycine 100 mM (3 washes of 15 min). Antigens were blocked with IF buffer (PBS, 7.7 mM NaN_3_, 0.1% bovine serum albumin, 0.2% Triton x-100, 0.05% Tween-20) + 10% goat serum for 1 h and then with IF buffer + goat serum + 20 µg/mL F(ab’) fragment (Jackson ImmunoResearch) for 30 min. Primary antibodies for K8 (TROMA, dshl, Developmental Studies Hybridoma Bank) and K5 (AF-138, Covance) were incubated overnight in a humid chamber. Fluorochrome-conjugated secondary antibodies (Invitrogen) were added later, diluted 1:500 in IF buffer +10% goat serum, and incubated for 40 min. Acini were then washed with IF buffer, cell nuclei were stained with DAPI (Sigma), and then mounted with Prolong ® Gold Antifade (Life Technologies). Confocal analysis was carried out using Leica confocal microscope. Images were captured using LasAF software (Leica).

### Statistics & reproducibility

Statistical analyses were performed using GraphPad Prism software version 8. Data are represented as the mean ± S.E.M. The measurements were taken from different mice, and we avoided technical replicates unless indicated. When comparisons were done between two experimental groups, an Unpaired, two-tailed Student’s *T*-Test was used. When comparing multiple variables between two experimental groups, an analysis of variance (ANOVA) was employed, followed by post hoc tests for multiple comparisons (Tukey). The statistical analyses of mRNA expression were conducted by comparing the experimental mice with their corresponding control. These analyses were conducted using a One-Sample *T*-Test, with a minimum of three independent mice. No statistical method was used to predetermine the sample size. In experiments where randomization was required (estradiol, MPA, prolactin, and Fc/RankFc treatment), block randomization was performed to maintain treatment groups even. In the lactation experiment and multiple pregnancies, we excluded female mice from the control group that lost their pups unexpectedly. Additionally, we excluded mice from the mid-pregnancy experiments if they were not found to be pregnant during necropsy. All investigators were blind to the time-point and treatment arms. Quantification of IF/IHC and data analysis was performed without knowledge of the identity of the samples.

### Reporting summary

Further information on research design is available in the Nature Portfolio Reporting Summary linked to this article.

### Supplementary information

Supplementary Information

Description of Additional Supplementary Files

Supplementary Data 1

Supplementary Data 2

Supplementary Data 3

Supplementary Data 4

Supplementary Data 5

Reporting Summary

### Source data

Source Data

## Data Availability

The raw data generated (RNA-seq experiments) in this study have been deposited in the Gene Expression Omnibus (GEO) database under accession code GSE199746. The mouse genome GRCm39 was used for the transcriptomic analyses (https://www.ncbi.nlm.nih.gov/grc/mouse/data). The signatures of mammary gland population profiles used in this study are available in the Gene Expression Omnibus (GEO) database under accession code GSE106273, GSE111113, GSE103275, GSE113197, GSE75688 and GSE149949. Source data are included with this paper. Biological materials can be obtained upon request. Source data are provided with this paper.

## References

[1] Watson CJ, Khaled WT (2020). Mammary development in the embryo and adult: new insights into the journey of morphogenesis and commitment. Development.

[2] Fata JE (2000). The osteoclast differentiation factor osteoprotegerin-ligand is essential for mammary gland development. Cell.

[3] Schramek D (2010). Osteoclast differentiation factor RankL controls development of progestin-driven mammary cancer. Nature.

[4] Beleut M (2010). Two distinct mechanisms underlie progesterone-induced proliferation in the mammary gland. Proc. Natl Acad. Sci..

[5] Joshi PA (2010). Progesterone induces adult mammary stem cell expansion. Nature.

[6] Joshi PA (2015). Rank signaling amplifies WNT-responsive mammary progenitors through R-SPONDIN1. Stem Cell Rep..

[7] Cordero A (2016). Rankl impairs lactogenic differentiation through inhibition of the Prolactin/Stat5 pathway at midgestation. Stem Cells.

[8] Benítez S (2021). Rank links senescence to stemness in the mammary epithelia, delaying tumor onset but increasing tumor aggressiveness. Dev. Cell.

[9] Pellegrini P (2013). Constitutive activation of Rank disrupts mammary cell fate leading to tumorigenesis. Stem Cells.

[10] Gonzalez-Suarez E (2007). Rank overexpression in transgenic mice with mouse mammary tumor virus promoter-controlled Rank increases proliferation and impairs alveolar differentiation in the mammary epithelia and disrupts lumen formation in cultured epithelial acini. Mol. Cell. Biol..

[11] Bach K (2017). Differentiation dynamics of mammary epithelial cells revealed by single-cell RNA sequencing. Nat. Commun..

[12] dos Santos CO, Dolzhenko E, Hodges E, Smith AD, Hannon GJ (2015). An epigenetic memory of pregnancy in the mouse mammary gland. Cell Rep..

[13] Feigman MJ (2020). Pregnancy reprograms the epigenome of mammary epithelial cells and blocks the development of premalignant lesions. Nat. Commun..

[14] Meier-Abt F (2013). Parity induces differentiation and reduces Wnt/Notch signaling ratio and proliferation potential of basal stem/progenitor cells isolated from mouse mammary epithelium. Breast Cancer Res..

[15] Lemay DG, Neville MC, Rudolph MC, Pollard KS, German JB (2007). Gene regulatory networks in lactation: identification of global principles using bioinformatics. BMC Syst. Biol..

[16] Centonze A (2020). Heterotypic cell-cell communication regulates glandular stem cell multipotency. Nature.

[17] Shackleton M (2006). Generation of a functional mammary gland from a single stem cell. Nature.

[18] Stingl J (2006). Purification and unique properties of mammary epithelial stem cells. Nature.

[19] Van Keymeulen A (2011). Distinct stem cells contribute to mammary gland development and maintenance. Nature.

[20] Prater MD (2014). Mammary stem cells have myoepithelial cell properties. Nat. Cell Biol..

[21] Saeki K (2021). Mammary cell gene expression atlas links epithelial cell remodeling events to breast carcinogenesis. Commun. Biol..

[22] Hanasoge Somasundara AV (2021). Parity-induced changes to mammary epithelial cells control NKT cell expansion and mammary oncogenesis. Cell Rep..

[23] Gonzalez-Suarez E (2010). Rank ligand mediates progestin-induced mammary epithelial proliferation and carcinogenesis. Nature.

[24] Pratt MAC (2009). The canonical NF-κB pathway is required for formation of luminal mammary neoplasias and is activated in the mammary progenitor population. Oncogene.

[25] van Weele LJ (2021). Depletion of Trp53 and Cdkn2a does not promote self-renewal in the mammary gland but amplifies proliferation induced by TNF-α. Stem Cell. Rep..

[26] Li CMC (2020). Aging-associated alterations in mammary epithelia and stroma revealed by Single-Cell RNA sequencing. Cell Rep..

[27] Hanada R (2009). Central control of fever and female body temperature by RankL/Rank. Nature.

[28] Tarutani M (1997). Tissue-specific knockout of the mouse Pig-a gene reveals important roles for GPI-anchored proteins in skin development. Proc. Natl Acad. Sci. Usa..

[29] Luque-Córdoba D, Priego-Capote F (2021). Fully automated method for quantitative determination of steroids in serum: An approach to evaluate steroidogenesis. Talanta.

[30] Smith T, Heger A, Sudbery I (2017). UMI-tools: modeling sequencing errors in Unique Molecular Identifiers to improve quantification accuracy. Genome Res.

[31] Langmead B, Salzberg SL (2012). Fast gapped-read alignment with Bowtie 2. Nat. Methods.

[32] Li H (2009). The sequence alignment/map format and SAMtools. Bioinformatics.

[33] Cock PJA, Fields CJ, Goto N, Heuer ML, Rice PM (2010). The Sanger FASTQ file format for sequences with quality scores, and the Solexa/Illumina FASTQ variants. Nucleic Acids Res..

[34] Quinlan AR, Hall IM (2010). BEDTools: a flexible suite of utilities for comparing genomic features. Bioinformatics.

[35] Graña O, Rubio-Camarillo M, Fdez-Riverola F, Pisano DG, Glez-Peña D (2017). Nextpresso: next generation sequencing expression analysis pipeline. Curr. Bioinform..

[36] Love MI, Huber W, Anders S (2014). Moderated estimation of fold change and dispersion for RNA-seq data with DESeq2. Genome Biol..

[37] Subramanian A (2005). Gene set enrichment analysis: a knowledge-based approach for interpreting genome-wide expression profiles. Proc. Natl Acad. Sci. USA..

[38] Debnath J, Walker SJ, Brugge JS (2003). Akt activation disrupts mammary acinar architecture and enhances proliferation in an mTOR-dependent manner. J. Cell Biol..

