# Supplementary material for: Luminal Rank loss decreases cell fitness leading to basal cell bipotency in parous mammary glands
Source: Nat Commun. 2023 Oct 9;14:6213. doi: 10.1038/s41467-023-41741-5 (PMC10562464; doi:10.1038/s41467-023-41741-5)
Supplement: Supplementary file 1 — Supplementary Information [file 41467_2023_41741_MOESM1_ESM.pdf]

# Supplementary Information

# Luminal Rank loss decreases cell fitness leading to basal cell bipotency in parous mammary glands

**Ana Sofia Rocha#, Alejandro Collado-Solé# et al.**

## Supplementary Figures 1-5

# Supplementary Figure 1

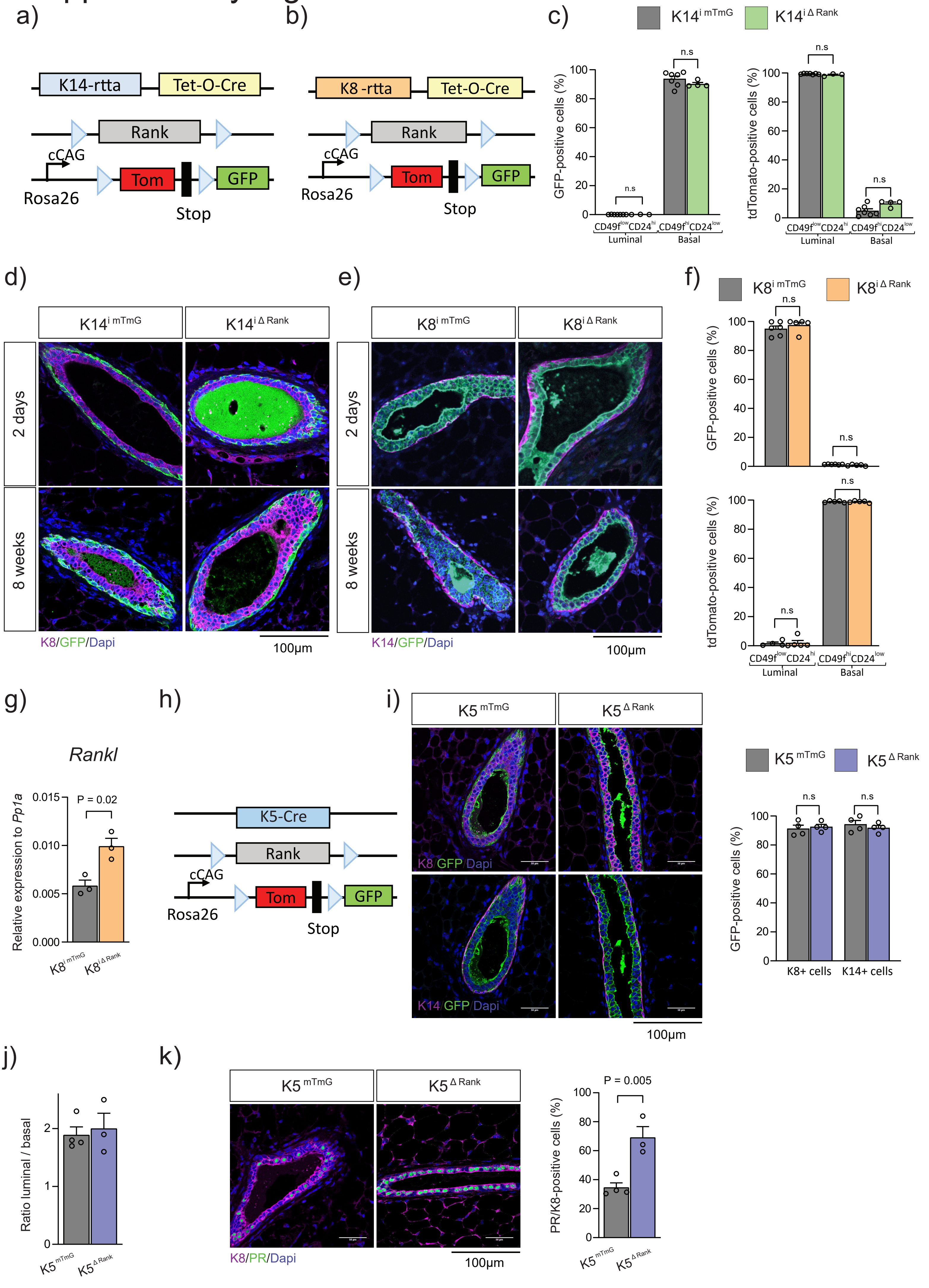

**Supplementary Figure 1: Epithelial Rank deletion reduces the luminal progenitor population without altering fat pad invasion. a**, **b** Genetic constructs of transgenic mouse models generated to delete Rank in basal ($K14^{i\ \Delta Rank}$ and control $K14^{i\ mTmG}$) or luminal ($K8^{i\ \Delta Rank}$ and control $K8^{i\ mTmG}$) cells and to trace control and Rank-depleted cells. **c** Flow cytometry analyses of GFP-positive cells and tdTomato-positive cells within the basal ($CD49f^{hi}/CD24^{low}$) and luminal ($CD49f^{low}/CD24^{hi}$) compartments in $K14^{i\ mTmG}$ (n=7) and $K14^{i\ \Delta Rank}$ (n = 4) 8 weeks post-dox removal. **d** IF analysis of K8 (magenta) and GFP (green) in $K14^{i\ mTmG}$ and $K14^{i\ \Delta Rank}$ MGs. **e** IF analysis of K14 (magenta) and GFP (green) in $K8^{i\ mTmG}$ and $K8^{i\ \Delta Rank}$ MGs two days and 8 weeks following dox removal. Dapi stains nuclei (blue). **f** Flow cytometry analyses of GFP-positive and tdTomato-positive cells within the basal ($CD49f^{hi}/CD24^{low}$) and luminal ($CD49f^{low}/CD24^{hi}$) compartments in $K8^{i\ mTmG}$ (n = 6) and $K8^{i\ \Delta Rank}$ (n = 5) 8 weeks post-dox removal. **g** *Rankl* qPCR analysis of flow cytometry sorted luminal cells in $K8^{imTmG}$ (n = 3) and $K8^{i\Delta Rank}$ virgin MGs. **h** Genetic construct of the transgenic model used to generate constitutive epithelial Rank deletion mouse model ($K5^{\Delta Rank}$ and control $K5^{mTmG}$). **i** IF analysis and quantification of recombination in luminal cells K8 (magenta)/GFP (green) and basal cells K14 (magenta)/GFP (green) in $K5^{\Delta Rank}$ (n = 4) and control $K5^{\ mTmG}$ (n = 4) mice (8 weeks). **j** Quantification of luminal (K8) to basal (K14) ratio measured by IF in $K5^{\ mTmG}$ (n=4) and $K5^{\Delta Rank}$ (n=3) virgin glands (8 weeks). **k** IF of PR (green) and K8 (magenta) and quantification of PR+ cells within the luminal population in $K5^{\ mTmG}$ (n=4) and $K5^{\Delta Rank}$ (n=3) virgin glands (8 weeks). Dapi (blue) stains nuclei (**c**, **d**, **g**, **i**). Data are represented as mean +/- SEM. Scale bars and significant *P* values are indicated in the graphs. *P* values were calculated by Two-Way ANOVA with Tukey's multiple comparisons (**c**, **f**, **i**) and Unpaired T-test two-tailed (**g**, **j**, **k**). Staining was quantified in 5 independent images from two tissues sections collected 100 µm apart (**i**, **j**, **k**). Source data are provided as a Source Data file. n.s = not significant.

# Supplementary Figure 2

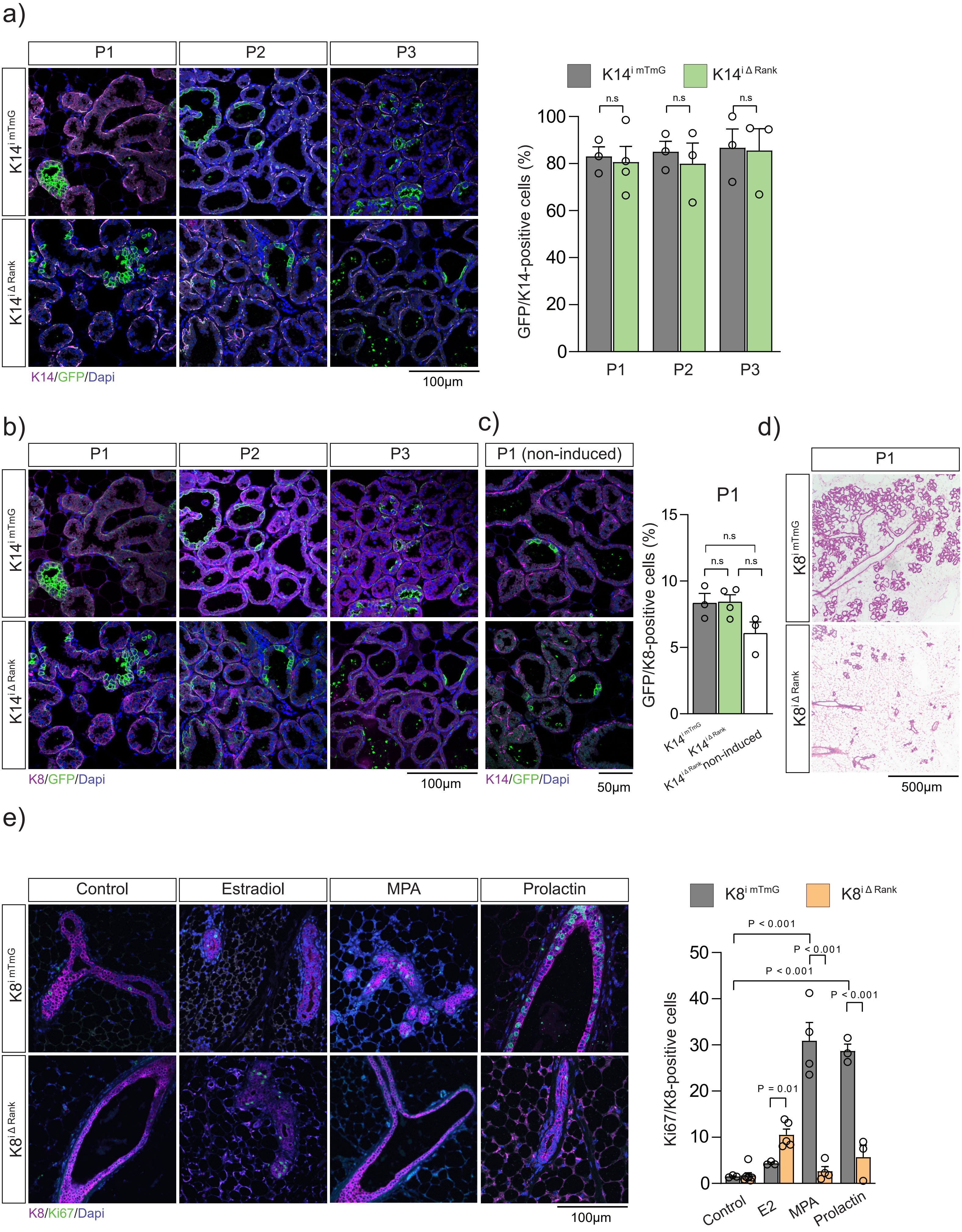

**Supplementary Figure 2: Luminal Rank is critical for functional lactogenic differentiation during pregnancy. a** IF analysis of K14 (magenta) and GFP (green) in K14$^{i\ mTmG}$ and K14$^{i\ \Delta Rank}$ MGs at L1 of P1, P2 and P3 and relative quantification of GFP/K14-positive cells in the indicated genotypes (n = 3). **b** IF analysis of K8 (magenta) and GFP (green) in K14$^{i\ mTmG}$ and K14$^{i\ \Delta Rank}$ MGs at L1 of P1, P2 and P3. **c** IF analysis of K14 (magenta) and GFP (green) in K14$^{i\ mTmG}$ and K14$^{i\ \Delta Rank}$ non-induced mice at L1 from P1. Quantification of GFP+ cells in the luminal population (K8+) of the indicated phenotypes at L1 from P1 compared to non-induced K14$^{i\ \Delta Rank}$ is shown (n = 3). **d** Haematoxylin-eosin stainings of mammary glands at L1 from K8$^{imTmG}$ and K8$^{i\Delta Rank}$ mice. **e** IF analysis of K8 (magenta) and Ki67 (green) in K8$^{imTmG}$ and K8$^{i\Delta Rank}$ MGs of virgin mice in control (n = 3 K8$^{imTmG}$ and n = 4 K8$^{i\Delta Rank}$ mice) and after treatment with estradiol (n = 3 K8$^{imTmG}$ and n = 5 K8$^{i\Delta Rank}$ mice), MPA (n = 4), or prolactin (n = 3). Quantification of Ki67 within the luminal compartment is shown. Dapi (blue) stains nuclei (**a**, **b**, **e**). Data are represented as mean +/- SEM. Scale bars and significant *P* values are indicated in the graphs. *P* values were calculated by Two-Way ANOVA with Tukey's multiple comparisons (**a**, **c, e**). Source data are provided as a Source Data file. n.s = not significant.

# Supplementary Figure 3

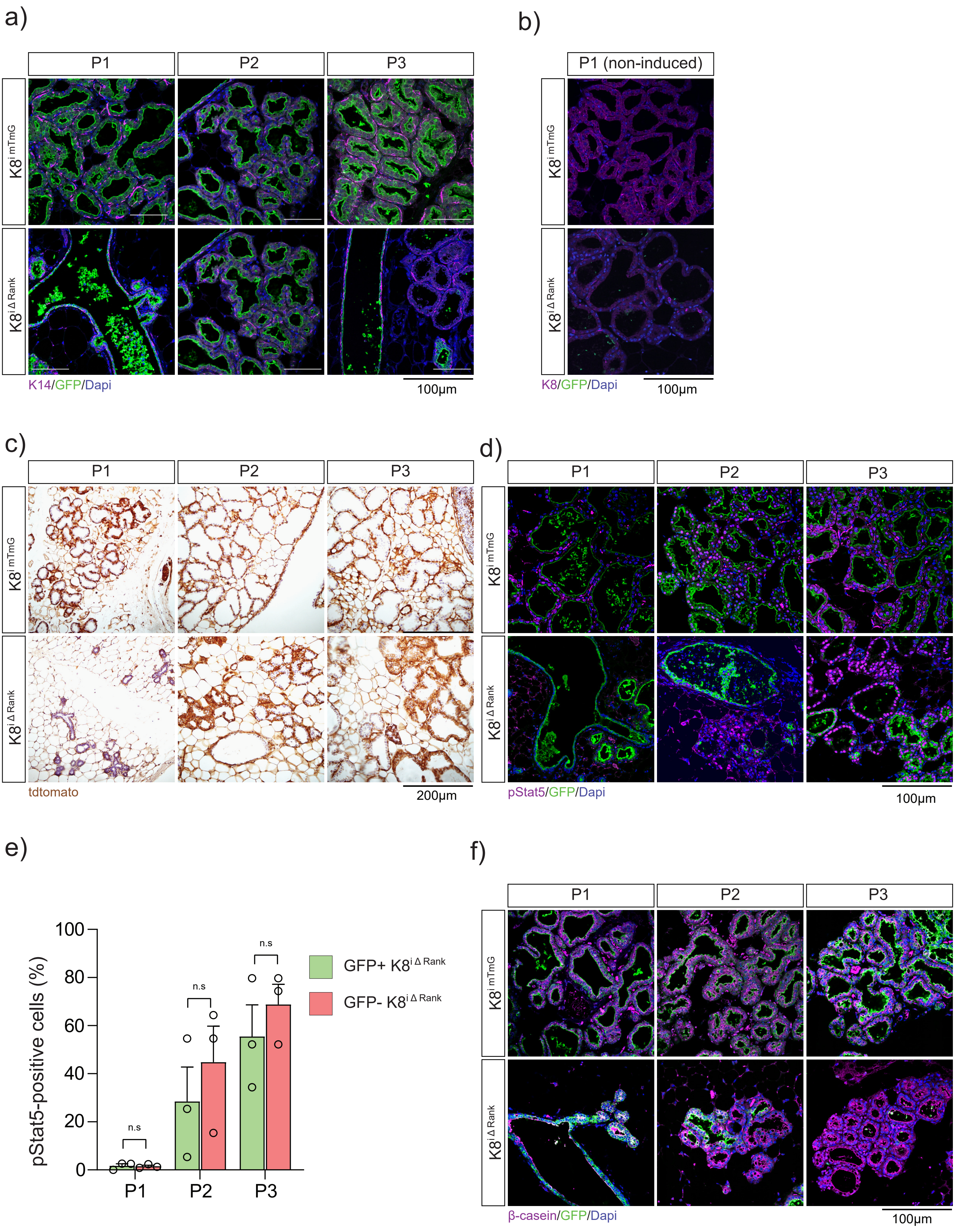

**Supplementary Figure 3: Luminal Rank loss leads to the emergence of Rank/tdTomato-positive cells following parity with activated Stat5 signaling. a** IF analysis of K14 (magenta) and GFP (green) in $K8^{i\ mTmG}$ and $K8^{i\ \Delta Rank}$ MGs at L1 of P1, P2 and P3. **b** IF analysis of K8 (magenta) and GFP (green) in $K8^{i\ mTmG}$ and $K8^{i\ \Delta Rank}$ non-induced mice at L1 from P1. **c** tdTomato IHC following P1, P2 and P3 at L1 in $K8^{imTmG}$ and $K8^{iRank}$ mice. **d** IF analysis of pStat5 (magenta) and GFP (green) in $K8^{i\ mTmG}$ and $K8^{i\ \Delta Rank}$ MGs at L1 of P1, P2 and P3. **e** Quantification of pStat5 in GFP+ and GFP- cells within the luminal compartment in $K8^{i\Delta Rank}$ MGs at L1 of P1, P2 and P3 (n = 3). **f** IF analysis of β-casein (magenta) and GFP (green) in $K8^{i\ mTmG}$ and $K8^{i\ \Delta Rank}$ MGs at L1 of P1, P2 and P3. Dapi (blue) stains nuclei (**a**, **b**, **d**, **f**). Data are represented as mean +/- SEM. Each dot represents a mouse. Scale bars and significant *P* values are indicated in the graphs. *P* values were calculated by Two-Way ANOVA with Tukey's multiple comparisons (**e**). Staining was quantified in 5 independent images from two tissues sections collected 100 µm apart (**e**). Source data are provided as a Source Data file. n.s = not significant.

# Supplementary Figure 4

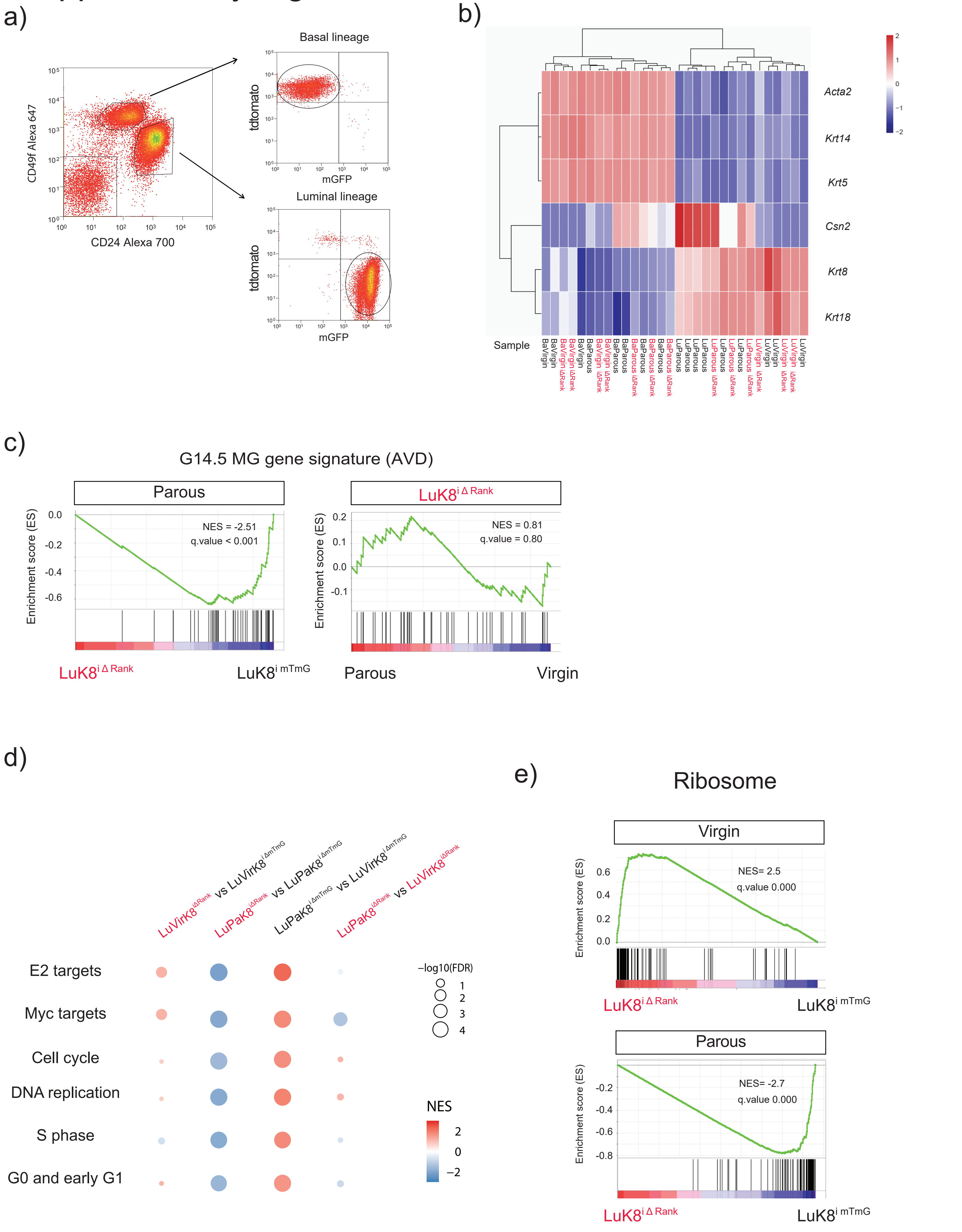

**Supplementary Figure 4: Rank deletion in luminal cells results in defective protein synthesis and proliferation upon parity. a** Flow cytometry cell sorting profile depicting the selection strategy for K8$^{i\ mTmG}$ and K8$^{i\ \Delta Rank}$ GFP+ luminal (CD49f$^{low}$ CD24$^{hi}$) and basal cells (CD49f$^{hi}$ CD24$^{low}$). **b** Heatmap of cell lineage specific genes to confirm the identity of the sorted cells analysed by RNA-seq (samples isolated from luminal K8$^{i\ \Delta Rank}$ mice are highlighted in magenta). **c** GSEA profile of alveolar progenitor differential gene set "G14.5 MG (AVD)" in the indicated genotypes. **d** Bubble plot depicting the variation of cell cycle related gene sets in luminal cells from virgin and parous K8$^{i\ mTmG}$ and K8$^{i\ \Delta Rank}$ mice. **e** GSEA profile depicting the differential regulation of "Ribosome" gene set in virgin and parous K8$^{imTmG}$ and K8$^{i\Delta Rank}$ luminal cells.

# Supplementary Figure 5

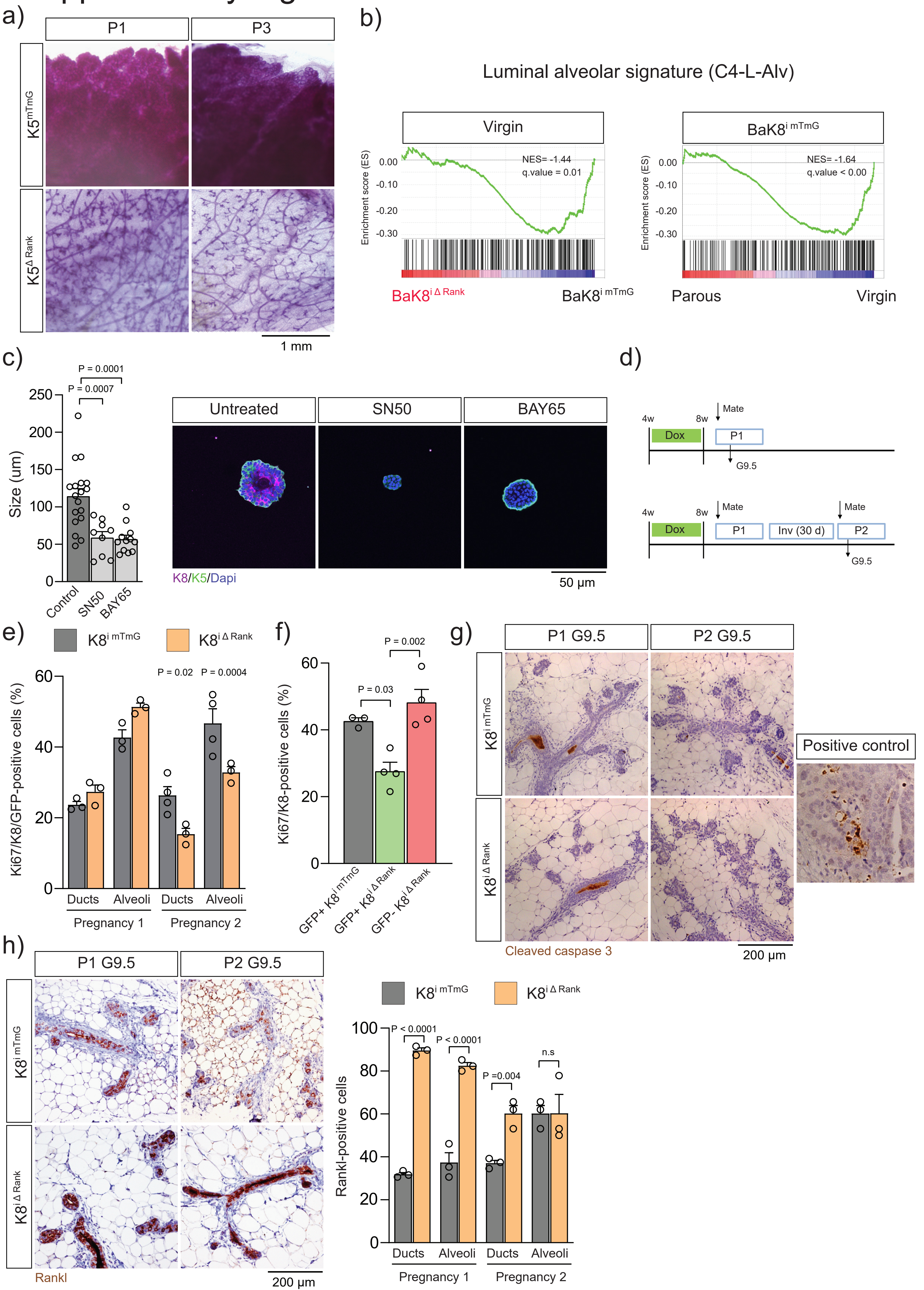

**Supplementary Figure 5: Rank/NF-κB activation is required for basal to luminal transition in parous MGs. a** Whole mount analysis (carmine aluminium staining) from $K5^{mTmG}$ and $K5^{\Delta Rank}$ MGs at L1 following P1 and P3. **b** GSEA profile of luminal alveolar identity gene set (C4-L-Alv) in virgin and parous Rank+ basal cells (Ba) of the indicated genotypes. **c** Size quantification of basal-derived organoids treated with NF-κB inhibitors SN50 (18 μM) and Bay65 (5 μM). Each dot represents an organoid of one of three independent experiments performed. Representative IF images for K8 (magenta) and K5 (green) in basal-derived mammary organoids treated with NF-κB inhibitors SN50 and Bay65 are shown. Dapi (blue) stains nuclei. **d** Protocol used to pinpoint the onset of GFP dilution in virgin and parous luminal glands in $K8^{imTmG}$ and $K8^{i\Delta Rank}$ mice. **e** Quantification of the proliferative index of recombined cells (Ki67+/K8+GFP+) in ducts and alveoli from $K8^{imTmG}$ and $K8^{i\Delta Rank}$ luminal cells at G9.5 from P1 (n = 3) and P2 (n = 4 $K8^{imTmG}$ and n = 3 $K8^{i\Delta Rank}$). **f** Quantification of the proliferative index (Ki67+) of GFP+/GFP- alveolar cells of $K8^{imTmG}$ (n = 3) and $K8^{i\Delta Rank}$ (n = 4) mice from an independent experiment. **g** Analysis of apoptosis (by cleaved caspase 3 IHC) in MGs at G9.5 from P1 and P2 of $K8^{imTmG}$ and $K8^{i\Delta Rank}$ mice. **h** Analysis of Rankl expression by IHC and quantification in ducts and alveoli of Rankl-positive cells at G9.5 from P1 and P2 of $K8^{imTmG}$ and $K8^{i\Delta Rank}$ mice (n = 3). Staining was quantified in 5 independent images from two tissues sections collected 100 μm apart (**e**, **f**, **h**). Source data are provided as a Source Data file. n. s = not significant.
