# Supplementary material for: Luminal Rank loss decreases cell fitness leading to basal cell bipotency in parous mammary glands
Source: Nat Commun. 2023 Oct 9;14:6213. doi: 10.1038/s41467-023-41741-5 (PMC10562464; doi:10.1038/s41467-023-41741-5)
Supplement: Supplementary file 2 — Description of Additional Supplementary Files [file 41467_2023_41741_MOESM2_ESM.pdf]

Description of Additional Supplementary Files

File Name: Supplementary Data 1
Description: **Hormonal analyses in serum:** Upper Table: Analysis of steroid hormones in the serum of mice at G14.5 using HPLC, comparing control (n=3) and K8iΔRank mice (n=3). P-values were calculated using Two-Way ANOVA with Tukey's multiple comparisons. Lower Table: Analysis of prolactin in the serum of mice at G14.5 using HPLC, comparing control (n=3) and K8iΔRank mice (n=3). A positive control of prolactin at L1 of a control mouse and prolactin-treated mice is presented.

File Name: Supplementary Data 2
Description: **Differentially expressed genes in luminal and basal K8iΔRank MECs:** List of genes that exhibit differential expression in luminal and basal cells from K8imTmG and K8iΔRank mice at both parous and virgin stages. To identify these DEGs, we utilized DESeq2 for differential expression analysis and normalization. Genes were retained for further analysis only if their normalized count value exceeded 10 in at least 25% of the samples. Hypothesis testing was performed using the Wald test, and p-value correction and false discovery rate (FDR) adjustment were carried out using the Benjamini-Hochberg (BH) method.

File Name: Supplementary Data 3
Description: **Gene set enrichment analyses (GSEA) of MGs signatures:** List of genes from the luminal alveolar differentiation signature at G14.5 (Bach et al 2017) and luminal alveolar signature (Saeki et al 2021).

File Name: Supplementary Data 4
Description: **Gene set enrichment analyses (GSEA) in luminal and basal K8iΔRank MECs:** GSEAPreranked was employed to conduct gene set enrichment analysis using selected gene signatures on a pre-ranked gene list, with 1000 gene set permutations performed. Correction of p-values and calculation of the false discovery rate (FDR) were carried out using the Benjamini-Hochberg (BH) method. The databases used for this analysis included Hallmark, Reactome, Biocarta, and KEGG.

File Name: Supplementary Data 5
Description: **Primers:** List of Forward and Reverse Primers used for qPCR Analyses
