# Supplementary material for: Luminal Rank loss decreases cell fitness leading to basal cell bipotency in parous mammary glands
Source: Nat Commun. 2023 Oct 9;14:6213. doi: 10.1038/s41467-023-41741-5 (PMC10562464; doi:10.1038/s41467-023-41741-5)
Supplement: Supplementary file 8 — Reporting Summary [file 41467_2023_41741_MOESM8_ESM.pdf]

nature portfolio

Corresponding author(s): Eva Gonzalez-Suarez
Ana Semiao Rocha

Last updated by author(s): 05-09-2023

Reporting Summary

Nature Portfolio wishes to improve the reproducibility of the work that we publish. This form provides structure for consistency and transparency in reporting. For further information on Nature Portfolio policies, see our Editorial Policies and the Editorial Policy Checklist.

Statistics

For all statistical analyses, confirm that the following items are present in the figure legend, table legend, main text, or Methods section.

n/a	Confirmed	
☐	☒	The exact sample size (*n*) for each experimental group/condition, given as a discrete number and unit of measurement
☐	☒	A statement on whether measurements were taken from distinct samples or whether the same sample was measured repeatedly
☐	☒	The statistical test(s) used AND whether they are one- or two-sided<br>*Only common tests should be described solely by name; describe more complex techniques in the Methods section.*
☒	☐	A description of all covariates tested
☒	☐	A description of any assumptions or corrections, such as tests of normality and adjustment for multiple comparisons
☐	☒	A full description of the statistical parameters including central tendency (e.g. means) or other basic estimates (e.g. regression coefficient) AND variation (e.g. standard deviation) or associated estimates of uncertainty (e.g. confidence intervals)
☐	☒	For null hypothesis testing, the test statistic (e.g. *F*, *t*, *r*) with confidence intervals, effect sizes, degrees of freedom and *P* value noted<br>*Give P values as exact values whenever suitable.*
☒	☐	For Bayesian analysis, information on the choice of priors and Markov chain Monte Carlo settings
☒	☐	For hierarchical and complex designs, identification of the appropriate level for tests and full reporting of outcomes
☒	☐	Estimates of effect sizes (e.g. Cohen's *d*, Pearson's *r*), indicating how they were calculated

Our web collection on statistics for biologists contains articles on many of the points above.

Software and code

Policy information about availability of computer code

Data collection	RNAseq data were feature extracted using QuantSeq 3‘mRNA-Seq Library Prep Kit (FWD) for Illumina
Data analysis	FASTQ files were analysed with the nextpresso pipeline (Graña et al., 2017). Sequencing quality was checked with FastQC v0.11.9. Software for RNAseq analyses:GSEA_4.2.3; R, BBDuk, Bowtie2 (version 2.4.2), DeepTools 3.5.0<br>Bubble plots were built with ggseabubble (https://gitlab.com/bu_cnio/ggseabubble).<br>BD FACSDiva v9.0 Software and FlowJo™ v10 were used to analyzed Flow cytometry data.<br>ImageJ 1.52o software was used to process images.

For manuscripts utilizing custom algorithms or software that are central to the research but not yet described in published literature, software must be made available to editors and reviewers. We strongly encourage code deposition in a community repository (e.g. GitHub). See the Nature Portfolio guidelines for submitting code & software for further information.

Data

Policy information about availability of data

All manuscripts must include a data availability statement. This statement should provide the following information, where applicable:

- Accession codes, unique identifiers, or web links for publicly available datasets
- A description of any restrictions on data availability
- For clinical datasets or third party data, please ensure that the statement adheres to our policy

The RNA-seq results obtained in this work have been deposited at GEO, GSE199746. The mouse genome GRCm39 was used for the transcriptomic analyses (https://www.ncbi.nlm.nih.gov/grc/mouse/data). Additional datasets are mentioned in the supplementary Figure S3, specifically the gene signatures of the MG profiles: GSE106273 (Bach et al. 2017), GSE111113 (Girradi et al. 2010), GSE103275 (Pal et al. 2011), GSE113197 (Human normal breast, Nguyen et al. 2017), GSE75688 (human breast cancer, Chung et al. 2016), and GSE149949 (Saeki et al. 2021)

Research involving human participants, their data, or biological material

Policy information about studies with human participants or human data. See also policy information about sex, gender (identity/presentation), and sexual orientation and race, ethnicity and racism.

Reporting on sex and gender	NA
Reporting on race, ethnicity, or other socially relevant groupings	NA
Population characteristics	NA
Recruitment	NA
Ethics oversight	NA

Note that full information on the approval of the study protocol must also be provided in the manuscript.

Field-specific reporting

Please select the one below that is the best fit for your research. If you are not sure, read the appropriate sections before making your selection.

☒ Life sciences ☐ Behavioural & social sciences ☐ Ecological, evolutionary & environmental sciences

For a reference copy of the document with all sections, see nature.com/documents/nr-reporting-summary-flat.pdf

Life sciences study design

All studies must disclose on these points even when the disclosure is negative.

Sample size	We designed our analyses of mouse samples to include a minimum of n ≥ 3 samples. Sample size determination was not based on statistical methods but rather on references from published authors in the fields of lineage tracing and mammary gland biology (DOI: 10.1038/nature10573, DOI: 10.1038/s41586-020-2632-y, DOI: 10.1128/MCB.01298-06).<br>For quantifying immunofluorescence (IF), we utilized five independent photos taken from two regions, each separated by 100 µm
Data exclusions	In the lactation experiment and multiple pregnancies, we excluded female mice from the control group that lost their pups unexpectedly. Additionally, we excluded mice from the mid-pregnancy experiments if they were not found to be pregnant during necropsy.
Replication	ELISA experiment and qPCR were performed in triplicate. The number of biological replicates is indicated in each experiment (figure legends).
Randomization	In experiments where randomization was required (estradiol, MPA, prolactin and Fc/RankFc treatment), block randomization was performed to maintain treatment groups even.
Blinding	All investigators were blind to the time-point and treatment arms. Quantification of IF/IHC and data analysis was performed without knowledge of the identity of the samples.

Reporting for specific materials, systems and methods

We require information from authors about some types of materials, experimental systems and methods used in many studies. Here, indicate whether each material, system or method listed is relevant to your study. If you are not sure if a list item applies to your research, read the appropriate section before selecting a response.

Materials & experimental systems

n/a	Involved in the study
☐	☒ Antibodies
☒	☐ Eukaryotic cell lines
☒	☐ Palaeontology and archaeology
☐	☒ Animals and other organisms
☒	☐ Clinical data
☒	☐ Dual use research of concern
☒	☐ Plants

Methods

n/a	Involved in the study
☒	☐ ChIP-seq
☐	☒ Flow cytometry
☒	☐ MRI-based neuroimaging

Antibodies

Antibodies used

The antibodies used for IHC on mouse tissue are:
mRankl (R&D, AF462). Dilution 1/100
Rank (R&D, AF692). Dilution 1/200
tdTomato (Rockland, 600-401-379). Dilution 1/100
cleaved Caspase 3 (Cell Signaling, #9661). Dilution 1/100

The antibodies used for IF on mouse tisse are:
K8 (obtained from the Developmental Studies Hybridoma Bank). Dilution 1/400
K14 (AF-64, Covance). Dilution 1/400
Ki67 (Abcam). Dilution 1/500
PR (SP2, 12683667, Fisher). Dilution 1/200
GFP (Abcam, ab13970). Dilution 1/500
pStat5 (9359S, Cell Signaling). Dilution 1/200
β-casein (Santa Cruz, sc-166530). Dilution 1/200

Secondary antibodies (Jackson Immunoresearch and FISHER SCIENTIFIC, S.L). Dilution 1/400: Goat anti-Rat IgG (H+L), Alexa Fluor 555; Donkey anti-Rabbit IgG Secondary Antibody, Alexa Fluor 555; Alexa Fluor 647-conjugated AffiniPure Donkey anti-chicken IgY (H+L), Alexa fluor 488 Donkey anti rabbit, Alexa fluor 555 Donkey anti goat.

Flow cytometry surface antibodies: CD24-alexa 700 (1.25 µg/mL, HMb1-1, BD Pharmingen), CD49f-alexa 647 (2.5 µg/mL, GoH3, BD Pharmingen), CD45-PECy7 (0.125 µg/mL, 30-F11 Biolegend) and CD31-PECy7 (0.5 µg/mL, 390 Biolegend).

Validation

FACs antibodies were tested and titrated following manufacturer's indications regarding concentration for flow cytometry and under the supervision of flow cytometry technicians at the Centres Científics i Tecnològics Universitat de Barcelona.

IHC and IF was performed using positive and negative controls. Validation of the antibodies are available in the website of each company.

Animals and other research organisms

Policy information about studies involving animals; ARRIVE guidelines recommended for reporting animal research, and Sex and Gender in Research

Laboratory animals

All research involving animals was performed at the IDIBELL and CNIO animal facilities in compliance with protocols approved by the corresponding Committee on Animal Care and following national and European Union regulations Mice were maintained in cages at animal facility (specific pathogen free, SPF) under controlled conditions of temperature (21 ± 1°C), humidity (55 ± 5%), cycles of light/dark of 12/12 h, and with food and water given ad libitum.

Rank flox/flox (Rankfl/fl) were provided by Dr. Joseph Penninger (Hanada et al., 2009) and crossed with either K14rtta:Tet-O-Cre or K8rtta:Tet-O-Cre, kindly provided by Cedric Blanpain, or K5-Cre (Tarutani et al., 1997). The resulting models were crossed with the reporter mouse line RosamT/mG (MGI 3716464) to perform lineage tracing analyses

The age of the mice varied depending on the specific experiments. For tracing experiments, the animal ages were specified in the figure legends as 8 weeks and 16 weeks. For experiments involving virgin mice, the age ranged from 8 to 20 weeks. For parous experiments, the mice were between 20 and 35 weeks old. In multiparous experiments (P2-P3), the animals' age ranged from 30 to 50 weeks

Wild animals

The study did not involved wild animals

Reporting on sex

As our study is focused on mammary gland development during pregnancy, we only used female mice.

Field-collected samples

The study did not involve field-collected samples

Ethics oversight

The study was conducted in accordance with the ethical guidelines established by the Comité de Ética en el Cuidado de Animales de la Comunidad de Madrid y la Comunidad Autónoma de Barcelona and in compliance with national and European Union regulations.

Specifically, all animal experiments were approved by our Institutional Animal Care and Use Committee (IACUC) and by the Ethical Committee for Animal Experimentation (CEIyBA) (PROEX028/19 and 161.2/21)

Note that full information on the approval of the study protocol must also be provided in the manuscript.

Flow Cytometry

Plots

Confirm that:

☒ The axis labels state the marker and fluorochrome used (e.g. CD4-FITC).

☒ The axis scales are clearly visible. Include numbers along axes only for bottom left plot of group (a 'group' is an analysis of identical markers).

☒ All plots are contour plots with outliers or pseudocolor plots.

☒ A numerical value for number of cells or percentage (with statistics) is provided.

Methodology

Sample preparation	Draining lymph nodes were removed and fresh mammary gland and were mechanically dissected with a McIlwain tissue chopper and enzymatically digested with appropriate medium (DMEM F-12, 0.3% Collagenase A, 2.5 U/mL dispase, 20 mM HEPES and antibiotics) for 40 min at 37°C. Samples were washed with Leibowitz L15 medium containing 10% fetal bovine serum (FBS) between each step. Erythrocytes were eliminated by treating samples with hypotonic lysis buffer (Lonza Iberica). Single cells were isolated by treating with trypsin (PAA Laboratories) for 2 min at 37°C. Cell aggregates were removed by filtering the cell suspension with a 70-µm filter and counted.<br>The marker is indicated in the axis, the fluorochrome used for each antibody is currently indicated in the methods section.
Instrument	MoFlo (Beckman Coulter)
Software	Diva software
Cell population abundance	The abundance of leukocytes ranged from 10% to 30%. Within the epithelial compartment (CD45-CD31-), luminal cells accounted for 60%, while basal cells comprised 40%.
Gating strategy	Firstly, scattering assists in distinguishing the population of interest by assessing its volume (forward scatter) and morphological complexity (side scatter). Subsequently, doublets and dead cells are removed from the analysis. Then, 10,000 CD45+ events are collected and classified into two populations: the luminal population (CD49flowCD24hi) and the basal population (CD49fhi/CD24low). Both populations are further examined for endogenous mtdtomato and mGFP expression.

☒ Tick this box to confirm that a figure exemplifying the gating strategy is provided in the Supplementary Information.
